# Risk-based Estimate of Effect of Foodborne Diseases on Public Health, Greece

**DOI:** 10.3201/eid1709.101766

**Published:** 2011-09

**Authors:** Elissavet Gkogka, Martine W. Reij, Arie H. Havelaar, Marcel H. Zwietering, Leon G.M. Gorris

**Affiliations:** Author affiliations: Wageningen University, Wageningen, the Netherlands (E. Gkogka, M.W. Reij, M.H. Zwietering, L.G.M. Gorris);; Utrecht University, Utrecht, the Netherlands (A.H. Havelaar);; National Institute for Public Health and the Environment, Bilthoven, the Netherlands (A.H. Havelaar);; Unilever Research and Development, Shanghai, People’s Republic of China (L.G.M. Gorris)

**Keywords:** enteric infections, parasites, bacteria, foodborne disease, disability-adjusted life year, DALY, risk ranking, food safety management, Greece, synopsis

## Abstract

The public health effects of illness caused by foodborne pathogens in Greece during 1996–2006 was quantified by using publicly available surveillance data, hospital statistics, and literature. Results were expressed as the incidence of different disease outcomes and as disability-adjusted life years (DALY), a health indicator combining illness and death estimates into a single metric. It has been estimated that each year ≈370,000 illnesses/million inhabitants are likely caused because of eating contaminated food; 900 of these illnesses are severe and 3 fatal, corresponding to 896 DALY/million inhabitants. Ill-defined intestinal infections accounted for the greatest part of reported cases and 27% of the DALY. Brucellosis, echinococcosis, salmonellosis, and toxoplasmosis were found to be the most common known causes of foodborne illnesses, being responsible for 70% of the DALY. Overall, the DALY metric provided a quantitative perspective on the impact of foodborne illness that may be useful for prioritizing food safety management targets.

## Abstract 2

TOC summary: These infections may account for 896 disability-adjusted life years per 1 million inhabitants annually.

To initiate and sustain efforts for prevention and control of foodborne diseases, it is essential to determine the extent and dimensions of the problem (*1*). Accurate knowledge of disease incidence and severity is invaluable to competent national authorities for use in selecting appropriate management actions to reduce the overall public health impact. However, much of the information collated regarding foodborne illnesses by different systems cannot be directly translated into policy (*2*) for 3 main reasons. First, not all cases are reported to health authorities, and estimates of underreporting result in considerable uncertainty in burden of illness studies, which limits the interpretation and analysis of available information (*3**,**4*). Second, often only a fraction of illnesses caused by food-related pathogens are actually foodborne because transmission can also be through the environment, direct contact with animals, or from person to person (*5*). Third, foodborne illnesses may vary not only in their incidence but also in their severity, resulting in widely different clinical manifestations and potentially involving long-term sequelae, although for their accurate description and quantification a uniform health measure would be needed (*6*).

To circumvent the latter issue, the World Health Organization (WHO) recommends using disability-adjusted life years (DALY) as a metric to express the public health effects of foodborne diseases (*2*), and DALY is increasingly used for a wide variety of illnesses (*6**–**8*). The aim of this study was to test the feasibility of using publicly available relevant data sources combined with the DALY metric to quantify the annual impact of foodborne illnesses in a country in a format useful for policy decisions. The country selected was Greece. The study used available surveillance data, hospital statistics from 1996 through 2006, and literature. In an attempt to address the first 2 limitations of the types of study mentioned above, we account in our estimates for uncertainty caused by underreporting and food attribution by using probability distributions to describe a range of plausible values for these parameters. Results are also expressed as cases in the general population, reported or estimated severe cases, and deaths to enable comparisons with similar studies in other countries.

## Methods

**Figure 1:**
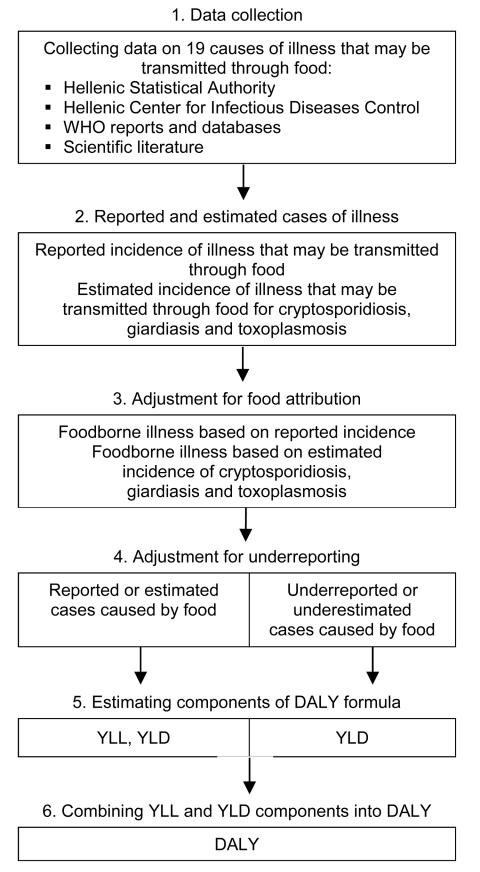
Working scheme for estimating the incidence and effects of foodborne illness in Greece. For cryptosporidiosis and giardiasis, because estimated cases are on the same level of the surveillance pyramid as reported cases, the cases occurring in the community (underestimated cases) were based on underreporting factors suitable for these pathogens. In the case of toxoplasmosis, disability-adjusted life years (DALY) are calculated only on the basis of estimated cases which cover the entire population. WHO, World Health Organization; YLL, years of life lost caused by premature death in the population; YLD, years lived with disability for incident cases of the health condition.

The various steps taken to estimate the incidence and impact of foodborne illness in Greece are shown in Figure 1. Reported cases of illnesses that may be transmitted through food were for the larger part collected from the Hellenic Statistical Authority (ELSTAT) (*9*) and the Hellenic Center for Infectious Diseases Control (HCIDC) (*10*). A limited number of data were obtained from WHO disease surveillance reports where HCIDC was mentioned to be the source (*11**,**12*) for better transparency and from other literature when no other information was available (*13*). The study included the period 1996–2006 for which data were available from both national sources. ELSTAT collects information regarding hospitalizations for case-patients who have a duration of stay >1 day based on the Basic Tabulation List (BTL) of the International Classification of Diseases, 9th Revision. ELSTAT data are based on sampling of hospitalized patients’ bulletins.

This sampling includes bulletins of deceased patients, although these bulletins are not recorded separately. Hospitalizations recorded by the ELSTAT are likely to vary in their severity because the population in Greece had free access to hospital centers where it was possible to be treated even for minor health issues (*14*). HCIDC collects information on notified cases from hospital microbiologic laboratories and district health authorities (*11*) and also performs active surveillance on the general incidence of gastroenteritis through physicians’ reports (*10*). HCIDC data can thus be representative of hospitalizations or visits to physicians and are a mixture of laboratory-confirmed and symptom-based notified cases. In the absence of a study validating these 2 systems of collecting information on disease incidence, we considered ELSTAT and HCIDC data to be representative of reported (severe) cases of illness. Corrections for undernotification or overnotification were not made because this would require a country-specific study that is not currently available. For the few illnesses for which data were available from both systems, ELSTAT data were preferred. For cryptosporidiosis, giardiasis, and toxoplasmosis, cases were estimated indirectly taking into account studies on prevalence of these parasites in the general population (*15**,**16*). The mean and standard deviation of reported and estimated cases for 1996–2006 were used to create normal distributions, which were considered representative of the annual incidence of these illnesses (*17*).

Deciding on a precise estimate of the proportion of cases that can be attributed to food is complicated (*5*). Because of differences in food production, consumption, and the ecology of pathogens, the percentage of foodborne transmission is expected to vary among countries and constitutes a major area of uncertainty. To make an adjustment for food attribution, PERT distributions were used as multipliers (*18*) (Table 1). Minimum and maximum parameters of PERT distributions were based on a literature search covering the range of potential values. Most likely values were based on data most relevant to Greece and Europe because endemicity of illnesses is often related to specific regions (*19**,**20*).

**Table 1:** Parameters of the PERT distributions used to describe foodborne transmission, underreporting, and case-fatality rate for foodborne illnesses, Greece, 1996–2006*

Illness	Minimum, most likely, maximum†		
	Food attribution, %	Underreporting	Case-fatality rate, %
Bacterial			
Botulism	80, 100, 100	1.625, 1.8125, 2	3, 10.15, 17.3
Brucellosis	50, 84,100	2, 10.85, 19.7	0.9, 2, 5
Campylobacteriosis	30, 55, 80	7.6, 274.8, 542	0.1, 0.1265, 0.153
Enterohemorrhagic *Escherichia coli*	40, 51, 90	2, 14.05, 26.1	0.25, 0.54, 0.83
Leptospirosis	1, 5, 49	10, 15, 20	5, 10, 15
Listeriosis	69, 99, 100	1.1, 1.7, 2.3	10, 30, 44
Salmonellosis	55, 95, 95	3.2, 51.45, 99.7	0.5, 0.701, 0.902
Shigellosis	8.2, 10, 31	3.4, 18.35, 33.3	0.1, 0.13, 0.16
Typhoid and paratyphoid fever	55, 80, 95	2, 7.65, 13.3	0.4, 0.95, 1.5
Food poisoning	87, 100, 100	29.3, 185.65, 342	0, 0.025, 0.05
Parasitic			
Amebiasis	10, 50, 100	9.2, 9.6, 10	0.1, 0.2, 0.3
Cryptosporidiosis	5.6, 5.6, 8	7.4, 53, 98.6	0.07, 0.335, 0.6
Echinococcosis	30, 30, 100	2, 3, 4	1, 2.24, 3
Giardiasis	5, 10, 30	4.6, 25.45, 46.3	0, 0.05, 0.1
Toxoplasmosis	30, 50, 63	NA	3.3, 3.75, 4.8
Viral: acute hepatitis A	5, 8, 11	2, 5.55, 9.1	0.3, 1.35, 2.4
Mixed/ill-defined causes			
Other helminthiases	30, 90, 100	4.6, 51.6, 98.6	3.37‡
Intestinal infections due to other specified microorganism	1, 36, 70	2, 402, 1,562	0.25‡
Ill-defined intestinal infections	1, 36, 50	2, 402, 1,562	0.0045‡

*NA, not applicable. †Minimum, most likely (mean), and maximum parameters of each PERT distribution. More information, including an expanded version of this table, can be found in the Technical Appendix. ‡For these illnesses, an average fixed value was used for the case-fatality rates estimated by using data from the World Health Organization Mortality Database on the deaths and incidence data from the Hellenic Statistical Authority.

Not all cases of foodborne illness are reported to health authorities (*3*), and the degree of underreporting varies greatly among diseases between countries or within 1 country in different periods (*21*). To make an adjustment for underreporting, PERT distributions were used as multipliers (*18*) and extremes were selected to cover the full range of values found in literature. Most likely values were set at the middle of this range to give equal weight to extremes of each distribution (Table 1). We assumed that underreporting factors primarily represent underreported cases for serious illnesses that result in physician visits, and underreporting factors for gastrointestinal illnesses are primarily associated with cases not resulting in physician visits. Although in some studies an arbitrarily assigned factor is used to cover for misdiagnosed or undiagnosed hospitalizations and deaths (*3**,**18*), it was omitted in the absence of specific data for Greece and underreported cases caused by this phenomenon were considered to be included in the “ill-defined intestinal infections” BTL code as suggested by other authors (*17*). We also assumed that all reported cases were diagnosed and coded correctly.

DALY values were calculated as DALY = YLL + YLD, where YLL are the years of life lost because of premature death in the population and YLD are the years lived with disability for incident cases of the health condition (*22*). YLD was estimated for reported or estimated cases and underreported cases, and YLL was estimated based only on reported or estimated cases. The rationale for this was that fatal cases contributing to YLL occur at the top of the surveillance pyramid and, if diagnosed, most likely are notified, particularly for obligatory notifiable diseases such as most of the ones examined here. Moreover, for illnesses contributing to YLD such as gastrointestinal illnesses, underreported cases not resulting in hospitalization are not expected to have fatal outcomes. The sole exception was listeriosis, in DALY values mainly accounted for through YLL (*23*), because it has been under surveillance only since 2004. Thus, even serious cases of this infection were expected to be considerably undernotified in part of the period under study because physicians and laboratories might not immediately be aware of the new reporting requirements. Therefore, to avoid underestimation of deaths, YLD for listeriosis was estimated on the basis of reported and underreported cases.

The individual components of the DALY formula are estimated as follows: YLL = *d* × *e*, where *d* is the number of deaths and *e* is the expected individual life span at the age of death in years; YLD = *n* × *t* × *w*, where *n* is the number of cases of a specific illness, *t* is its duration in years and *w* is a weight factor (disability weight) that reflects its severity on a scale from 0 (perfect health) to 1 (death) (*22**,**24*). In calculating YLL, the number of deaths (*d*) was estimated by multiplying reported or estimated cases caused by foodborne infection for each illness with a PERT distribution describing a plausible range of pathogen-specific case-fatality rates (*18*) on the basis of literature data from other industrialized countries (Table 1). Selected case-fatality rates were always from the same level of the surveillance pyramid as reported for estimated cases. For some generic BTL codes (e.g., “Other helminthiases,” “Intestinal infections due to other specified microorganism,” and “Ill-defined intestinal infections”), the number of deaths was based on data from the WHO Mortality Database (*25*).

Regarding the expected individual life span at the age of death in years (*e*), the age of death was estimated on the basis of data collected by the HCIDC and ELSTAT on patients’ age in reported cases. When no explicit information was available in these sources, which was the case for 5 illnesses, age at time of death was assumed to be 40 years. To check the impact of this assumption on the ranking of foodborne risks, we tested both extremes by assuming 0 years as the age at death and by assuming YLL to be 0. For “Other helminthiases,” data from the WHO Mortality Database were used. General life expectancy was based on the life table for Greece for 2000 (*22*). For comparison, estimates were also made by using the WHO standard West Level 26 life table (*22*).

In calculating YLD, duration of illness (*t*) was based on data collected by ELSTAT and on literature regarding serious and mild forms of each cause of illness. Different disability weights (*w*) were used for each disease based on the severity of its sequelae and whether estimated cases likely reach the health system or not (Table 2). All underreported cases were assumed to be mild or self-limiting for gastroenteritis-related illnesses. For serious, non–self-limiting diseases such as brucellosis or echinococcosis that are not related to gastroenteritis, nonreported cases were considered to be as severe as reported or estimated cases.

**Table 2:** Disability weights related to the diseases included in study of the effects of foodborne infections, Greece, 1996–2006

Illness	Disability weights	
	Reported or estimated cases	Underreported cases
Bacterial		
Botulism		
Moderate cases	0.600	0.600
Severe cases	0.906	0.906
Brucellosis	0.200	0.200
Campylobacteriosis		0.067
Gastroenteritis	0.393	
Reactive arthritis	0.140	
Guillain-Barré syndrome, first year*	0.250	
Guillain-Barré syndrome, long-term sequelae	0.160	
Inflammatory bowel disease	0.260	
Irritable bowel syndrome	0.042	
Enterohemorrhagic *Escherichia coli*		0.067
Watery diarrhea and hemorrhagic colitis	0.393	
Hemolytic uremic syndrome and end-stage renal disease	†	
Leptospirosis	0.920	0.096
Listeriosis	‡	‡
Salmonellosis		0.067
Gastroenteritis	0.393	
Inflammatory bowel disease	0.260	
Irritable bowel syndrome	0.042	
Reactive arthritis	0.150	
Shigellosis	0.220	0.096
Irritable bowel syndrome	0.042	
Typhoid and paratyphoid fever	0.600	0.096
Food poisoning	0.220	0.067
Parasitic		
Amebiasis	0.400	0.067
Cryptosporidiosis	0.393	0.067
Echinococcosis		
Cured	0.200	0.200
Postsurgical conditions	0.239	0.239
Relapse	0.809	0.809
Undiagnosed	0.200	0.200
Giardiasis	0.393	0.067
Toxoplasmosis		
Clinical symptoms in the first year of life§	0.140	¶
Asymptomatic at birth, chorioretinitis later in life	0.080	¶
Viral: acute hepatitis A	0.500	0.500
Mixed/ill-defined causes		
Other helminthiases	0.463	0.067
Intestinal infections caused by other specified microorganism	0.400	0.067
Ill-defined intestinal infections	0.400	0.067

*For an explanation of this selection, see the Technical Appendix. †For hemolytic uremic syndrome (including end-stage renal disease as a sequela), it is estimated that every case corresponds to 1.05 years lived with disability (*24*). ‡Not applicable for listeriosis because the high case-fatality rate (>95%) of the disability-adjusted life year estimates is composed of years of life lost (*24*) that mainly determine the burden of the disease. Therefore, no years lived with disability were estimated. §Clinical symptoms in the first year of life include chorioretinitis, intracranial calcifications, hydrocephalus, and central nervous system abnormalities that lead to neurologic deficiencies such as mental retardation. ¶Toxoplasmosis cases are estimates for the entire population. Consequently, underreporting does not apply.

All estimations were performed by using the @RISK 5.7 software (Palisade Corporation, Ithaca, NY, USA) as an add-in in Microsoft Excel 2010 (Microsoft, Redmond, WA, USA). Full details regarding estimations of DALY, selection of input distributions and simulation settings can be found in the Technical Appendix.

## Results

### Annual Incidence of Foodborne Illnesses

For 1996–2006, we estimated 369,305 (95% credible interval [CrI] 68,283–910,608) illnesses per million inhabitants per year attributable to eating contaminated food, at least 905 of which (95% CrI 499–1,340) are reported or estimated to be severe and 3 fatal (95% CrI 2.0–4.8) (Table 3). Ill-defined intestinal infections accounted for most (94%) cases (sum of reported/estimated and underreported cases). Regarding reported/estimated cases, ill-defined intestinal infections were responsible again for the greatest part (72%), followed by salmonellosis (8.2%), brucellosis (7.1%), food poisoning (4.0%), and echinococcosis (2.7%). Most deaths (48%) were estimated to be caused by brucellosis, although salmonellosis, echinococcosis, listeriosis, and toxoplasmosis also contributed substantially to deaths.

**Table 3:** Mean estimated incidence of total foodborne illnesses, reported/estimated illnesses, and deaths attributed to food in Greece per 1 million inhabitants, 1996–2006*

Illnesses	Incidence per million inhabitants							
	Total illnesses			Reported/estimated illnesses			Deaths	
Mean†	95% CrI‡	Mean†	95% CrI‡	Mean†	95% CrI‡
Bacterial								
Botulism	0.13	0.011–0.28		0.066	0.056–0.15		0.0067	0.00052–0.017
Brucellosis	699	225–1,378		**64**	30–102		**1.5**	0.52–3.0
Campylobacteriosis	**3,571**	851–7,733		13	5.6–22		0.016	0.0069–0.029
EHEC	1.0	0.069–2.8		0.072	0.0058–0.17		0.00039	0.000030–0.00098
Leptospirosis	4.0	0.34–13		0.27	0.023–0.84		0.027	0.0022–0.087
Listeriosis	0.89	0.11–1.9		0.41	0.049–0.85		**0.19**	0.021–0.45
Salmonellosis	**3,793**	750–8,350		**74**	22–128		**0.52**	0.15–0.93
Shigellosis	25	1.1–77		1.4	0.068–3.8		0.0018	0.000088–0.0050
Typhoid and paratyphoid fever	37	3.3–92		4.8	0.47–10		0.046	0.0043–0.11
Food poisoning	**6,636**	450–17,569		**36**	2.8–80		0.0089	0.00055–0.025
Parasitic								
Amebiasis	13	1.9–29		1.3	0.19–3.0		0.0026	0.00037–0.0064
Cryptosporidiosis	197	71–360		3.7	2.4–5.3		0.013	0.0050–0.022
Echinococcosis	72	29–140		**24**	10–45		**0.52**	0.19–1.0
Giardiasis	159	47–358		6.3	2.7–12		0.0031	0.00069–0.0074
Toxoplasmosis	3.4	2.5–4.1		3.2	2.4–4.0		**0.12**	0.090–0.16
Other helminthiases	137	22–322		2.7	0.56–5.1		0.089	0.019–0.17
Viral: hepatitis A	6.9	1.4–15		1.2	0.27–2.4		0.017	0.0031–0.038
Mixed/ill-defined causes								
Intestinal infections caused by other specified microorganism	**7,394**	354–25,558		14	1.2–36		0.035	0.031–0.091
Ill-defined intestinal infections	**346,558**	45,985–886,276		**655**	256–1,082		0.030	0.012–0.049
Total of gastroenteritis	368,520	67,536–909,457		812	408–1,245		0.95	0.52–1.4
Total	369,305	68,283–910,608		905	499–1,340		3.1	2.0–4.8

*Values have been rounded to include significant digits and thus not all summations necessarily tally. **Boldface** indicates the top 5 contributors to each estimate category. EHEC, enterohemorrhagic *Escherichia coli*; CrI, credible interval. †These estimates correspond to the mean of the output distributions. ‡95% CrI representative of the 2.5 and 97.5 percentiles.

### Public Health Impact of Foodborne Illnesses Expressed as DALY

**Figure 2:**
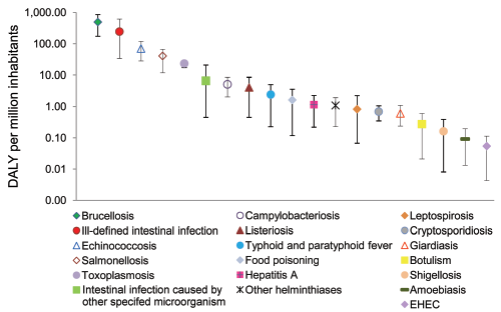
Disability-adjusted life years (DALY) caused by different foodborne diseases per million inhabitants in the course of an average year in Greece, including uncertainty. Estimates are presented on a logarithmic scale on the y-axis. Whiskers represent 95% credible intervals. EHEC, enterohemorrhagic *Escherichia coli*.

Foodborne illnesses accounted for ≈896 DALY per 1 million inhabitants annually (95% CrI 470–1,461), of which 14% were attributable to YLL and 86% to YLD (Table 4). As much as 34% of the estimated effects of foodborne disease in Greece could be attributed to gastroenteritis-related illnesses, and the remaining 66% was unevenly split among 6 non–gastroenteritis-related illnesses (brucellosis, echinococcosis, toxoplasmosis, leptospirosis, hepatitis A, and botulism). Notwithstanding attendant uncertainty (Figure 2), the most serious foodborne illness in Greece was brucellosis, representing ≈55% of the estimated DALY and contributing greatly to illness (>88%). Ill-defined intestinal infections were the second most serious contributor to disease burden (≈27% of DALY), followed by echinococcosis (7.8%) and salmonellosis (4.6%) as known causes of illness.

**Table 4:** Estimates of YLL, YLD, and DALY caused by foodborne illnesses in an average year in Greece per 1 million inhabitants, including plausible range attributable to uncertainty*

Illnesses	Estimated YLL (95% CrI)†	Estimated YLD (95% CrI)†	Estimated DALY (95% CrI)†
Bacterial			
Botulism	0.27 (0.021–0.67)	0.0066 (0.00056–0.015)	0.28 (0.021–0.69)
Brucellosis	**59 (21–121)**	**434 (140–856)**	**493 (174–943)**
Campylobacteriosis	1.2 (0.51–2.1)	3.9 (1.5–7.5)	5.14 (2.0–9.4)
Enterohemorrhagic *Escherichia coli*	0.016 (0.0012–0.039)	0.039 (0.0031–0.091)	0.054 (0.0043–0.13)
Leptospirosis	0.81 (0.066–2.7)	0.015 (0.0013–0.046)	0.83 (0.067–2.7)
Listeriosis	**4.1 (0.45–9.7)**	‡	4.1 (0.45–9.7)
Salmonellosis	**31 (8.7–55)**	**10 (2.9–19)**	**41 (12–72)**
Shigellosis	0.12 (0.060–0.34)	4.1 (0.0021–0.12)	0.16 (0.0081–0.46)
Typhoid and paratyphoid fever	2.3 (0.21–5.4)	0.17 (0.016–0.38)	2.4 (0.23–5.7)
Food poisoning	0.36 (0.022–0.98)	1.3 (0.088–3.3)	1.6 (0.12–4.1)
Parasitic			
Amebiasis	0.079 (0.011–0.20)	0.013 (0.0019–0.030)	0.092 (0.013–0.22)
Cryptosporidiosis	0.50 (0.20–0.88)	0.20 (0.10–0.32)	0.69 (0.35–1.2)
Echinococcosis	**16 (5.9–31)**	**54 (22–106)**	**70 (28–135)**
Giardiasis	0.12 (0.028–0.29)	0.48 (0.18–0.99)	0.61 (0.24–1.2)
Toxoplasmosis	**9.7 (7.0 −13)**	**14 (10–17)**	**23 (17–29)**
Other helminthiases	0.92 0.19–1.8)	0.17 (0.029–0.38)	1.1 (0.23–2.1)
Viral: hepatitis A	1.1 (0.20–2.4)	0.089 (0.018–0.19)	1.2 (0.22–2.6)
Mixed/ill-defined causes			
Intestinal infections caused by other specified microorganism	1.4 (0.12–3.6)	5.2 (0.26–18.0)	6.6 (0.45–21)
Ill-defined intestinal infections	1.2 (0.5–2.0)	**243 (33–621)**	**245 (34–622)**
Total of gastroenteritis§	43 (20–68)	265 (55–643)	308 (94–687)
Total	130 (81–196)	767 (361–1,308)	896 (470–1,461)

*Values have been rounded to include significant digits and thus not all summations necessarily tally. **Boldface** indicates the top 5 contributors to each estimate category. YLL, years of life lost; YLD, years lived with disability; DALY, disability-adjusted life years; CrI, credible interval. †95% CrI representative of the 2.5 and 97.5 percentiles. ‡DALY due to listeriosis are mainly determined by the YLL (*23*); therefore, no YLD were estimated. §Gastroenteritis-related illnesses are considered to be all of the above except: botulism, brucellosis, leptospirosis, echinococcosis, hepatitis A, and toxoplasmosis.

## Discussion

The DALY metric provided a different view on the effects of foodborne illnesses on public health in comparison to incidence estimates (Table 5). Although salmonellosis was captured as a major contributor by all 4 rankings, there was variation regarding other causes of illness. Interestingly, diseases that have the highest effect on public health either in terms of illness (ill-defined intestinal infections), death (toxoplasmosis) or both (brucellosis) are not identified in the ranking based on a single individual incidence parameter, but they are captured by DALY, which has the advantage of enabling comparisons between different disease endpoints. For instance, although toxoplasmosis is not among the 5 major contributors on the basis of the total incidence or on reported/estimated cases, it is given more prominence through using the DALY metric because this also accounts for severe outcomes and sequelae of this disease. Although self-limiting diseases may appear to be essential in terms of incidence, on the basis of DALY they do not greatly contribute to either illness or death. Therefore, use of the DALY metric gives a different and risk-based perspective of the influence of foodborne illnesses on the health of a country’s population because it is estimated on the basis of the diseases’ frequency (incidence) and severity (health effect).

**Table 5:** Ranking of the top 5 causes contributing to the effects of foodborne illness in Greece as estimated on the basis of individual incidence parameters and disability-adjusted life years, 1996–2006

Rank	Incidence estimates			Disability-adjusted life years
All foodborne illnesses	Reported/estimated illnesses	Deaths
1	Ill-defined intestinal infections	Ill-defined intestinal infections	Brucellosis	Brucellosis
2	Intestinal infections due to other specified causes	Salmonellosis	Salmonellosis	Ill-defined intestinal infections
3	Food poisoning	Brucellosis	Echinococcosis	Echinococcosis
4	Salmonellosis	Food poisoning	Listeriosis	Salmonellosis
5	Campylobacteriosis	Echinococcosis	Toxoplasmosis	Toxoplasmosis

Most of the foodborne illness cases in Greece were caused by ill-defined intestinal infections (Table 3). This finding is consistent with results from similar studies in other countries (*3**,**17*). Using the current Greek surveillance system, we cannot attribute this burden to known causes of gastroenteritis other than the ones included in this study. Noroviruses could be the etiologic agents in a large proportion of these ill-defined intestinal infections because they have been considered the most likely agent of foodborne illness caused by unknown agents (*26*) and have been found in other studies to be a most common cause of foodborne illness due to known agents (*17**,**18*). Outbreak data found for these pathogens were scarce (*27*) and therefore not included in this study. A considerable part of this category might also have been caused by other unknown agents of illness or known agents that have been misdiagnosed. For instance, campylobacteriosis is expected to be undiagnosed to a great extent in Greece because few laboratories in the country have the ability to identify the pathogen (*10*). This finding could partially explain the high underreporting factor estimated for this illness for Greece, based on the approach of Ekdahl and Giesecke (*28*) compared with results for other Western countries (*3**,**29*).

**Figure 3:**
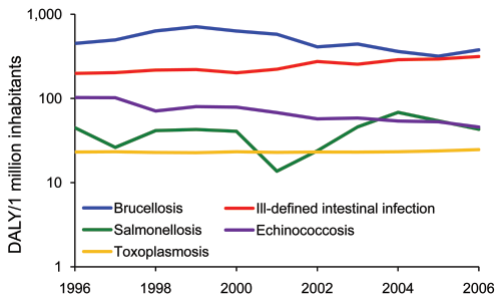
Trends for the top 5 contributors to the burden of foodborne diseases in Greece, 1996–2006. DALY, disability-adjusted life years.

Brucellosis was found to be the leading cause of illness and death in Greece. Although its incidence showed a reasonably consistent decline during the period of this study, it still constitutes a serious public health problem (Figure 3). The disease is most common in rural areas of the country, and risk factors for its contraction are occupational contact with animals and the consumption of unpasteurized milk and milk products (*30**,**31*).

Echinococcosis was the second most notable foodborne illness. This disease has been recognized as a serious health problem in the country (*32*) and linked with contaminated food (*10**,**33*). Echinococcosis caused by *Echinococcus granulosus* (cystic echinococcosis) is the dominant form in Greece (*32*), where the infection is hyperendemic (*19*). Although its incidence has gradually decreased since 1984 as a result of a long anti-echinococcosis campaign and general improvements in living and hygiene standards (*32*), it still is a serious health risk for the population (Figure 3).

Salmonellosis was the third most serious foodborne illness of known etiology in terms of public health impact, and it also was the most prominent gastroenteritis-related illness of identified cause (Table 3). This finding is consistent with it being a noteworthy zoonosis, which contributes to a high prevalence of gastrointestinal illness in the European Union (*34*), and the most often reported causative agent of outbreaks of an identified etiologic agent worldwide (*35*).

After salmonellosis, congenital toxoplasmosis was also a major contributor to the disease burden, although in terms of incidence it is an uncommon illness with <4 cases per million inhabitants. The disease has not been recognized as a major foodborne illness in the country, although its serious health consequences have been well documented (*36*).

There are 4 major factors that add to the uncertainty in our estimates that are not independent: 1) underreporting, 2) food attribution, 3) the quality of incidence data, and 4) value choices in the DALY formula. Given the limited data available for Greece, data from other countries have been used to create multipliers for underreporting and foodborne transmission (Technical Appendix); these data were of variable quality and representativeness. For instance, in the case of campylobacteriosis and salmonellosis, underreporting factors based on tourist studies (*28**,**37*) have been included in the multipliers, which were higher than underreporting factors from other Western countries for the same pathogens (*4**,**18*). Such underreporting factors might not be completely representative of the difference between reported cases resulting in physician visits and cases in the general population because these studies can be subject to several biases (e.g., tourists differ from natives in exposure) (*28*), although at the same time they cover for phenomena such as undernotification and misdiagnosis of illnesses that were beyond our intention. As a consequence of including data derived by using different method approaches, the plausible range of these multipliers was wide, which resulted in DALY estimates with similarly wide credible intervals (Figure 2). However, despite this limitation, our estimates can still be used for risk ranking purposes.

Uncertainty is also an inherent property of incidence data. Specifically, data for reported cases in Greece (and elsewhere) rely on insufficiently detailed codes, there is incomplete or lacking separate surveillance for many foodborne pathogens, and a specific diagnosis is not given for most episodes of enteric illness requiring hospitalization. These factors result in the greater part of reported cases of gastroenteritis being attributed to ill-defined causes. As with other studies of this kind, assumptions had to be made, notably considering the age of death. Although this assumption did not change the 5 major foodborne risks, it had considerable impact on the individual estimates. We also had to assume that serious cases of illness that have been reported because of a specific agent have been diagnosed and coded correctly or notified to the appropriate authorities. This assumption might not always be the case because at least some of these illnesses are expected to be part of the ill-defined illnesses. A correction for misdiagnosis and undernotification cannot be included for the reported illnesses until country specific data are available. Assigning an arbitrary factor as in other studies (*3*) introduces new uncertainties and, unlike incidence data in the case of DALY, can affect the ranking of foodborne risks. Thus, our estimates are based only on the illnesses that the surveillance system in Greece currently exposes, and the estimates’ robustness can only be further improved through improved surveillance.

As for uncertainty resulting from value choices in the DALY formula itself, in the present study no age-weighting or discounting were used because their combined use has been criticized as attributing considerably fewer disease impacts and effects to younger age groups (*38*), and disability weights were carefully selected. For policy-making purposes, ideally, disability weights should be based on the opinion of the general public because they should reflect preferences of the society being studied (*21*). Conceivably, use of the DALY metric could help reduce a considerable part of overall uncertainty by accounting for sequelae, which are not normally taken into consideration in studies focusing solely on incidence of foodborne illness yet do constitute a substantial part of the overall effects on a population. In our study, all well-defined sequelae for which information existed in literature were used for DALY calculations, but our findings could be subject to change when new insights become publicly available. For instance, rates of posthospitalization morbidity related to gastrointestinal illnesses have not been taken into account in the absence of a specific study, although the duration of illness can be longer than the actual hospital stay.

Finally, selection of life tables is another factor that can influence the DALY estimates. When our estimates could be based on West Level 26 life tables, total burden of illness expressed as DALY increased by only 0.0042%, although individual estimates for illnesses could differ by up to 5.0% (results not shown).

Regarding the total incidence of foodborne illnesses, our estimates were in the same range as the estimates for Australia (Table 6), although somewhat higher because the study by Hall et al. was restricted to gastroenteritis-related foodborne illnesses (*17*). Our estimates of severe reported or estimated cases are between the range of hospitalization rates mentioned for different countries, and the same is the case for our case-fatality rates. Our DALY estimates were higher than estimates for the Netherlands (*7*) or New Zealand (*39*), although our estimated overall impact for gastrointestinal illnesses is still comparable to the one from the Netherlands where brucellosis is not a major foodborne risk.

**Table 6:** Comparison of foodborne illness effects on public health in Greece with estimates from other countries*

Country (reference)	Target	Disease estimates per 1 million inhabitants			
		All illnesses†	Hospitalizations	Deaths	DALY
United States (*3*)	All causes	270,057	1,155	18	NA
United States (*18*)	Known agents	31,438 (90% CrI 22,074–42,475)	187 (90% CrI 132–253)	5 (90% CrI 2–8)	NA
United States (*40*)	Unspecified agents	128,404 (90% CrI 66,318–204,670)	240 (90% CrI 33–526)	6 (90% CrI 1–11)	NA
England and Wales (*29*)	All causes	26,161	406	9	NA
Australia (*17*)	Gastro	281,250 (95% CrI 208,333–359,375)	766 (95% CrI 594–922)	4 (95% CrI 2–6)	NA
The Netherlands (*7*)	All causes	79,725–104,256	NA	1–12	184–613
New Zealand (*39*)	6 agents‡	128,421 (95% CrI 34,801–330,075)	NA	NA	632 (95% CrI 344–1,066)
Greece (this study)	All causes	369,305 (95% CrI 68,283–910,608)	905 (95% CrI 499–1,340)§	3.1 (95% CrI 2.0–4.8)	896 (95% CrI 470–1,461)
Greece (this study)	Gastro only	368,520 (95% CrI 67,536–909,457)	812 (95% CrI 408–1,245)§	0.95 (95% CrI 0.52–1.4)	308 (95% CrI 94–687)

*Data have been normalized for population differences and are expressed per million inhabitants. DALY, disability-adjusted life years; NA, not available; CrI, credible interval; gastro, gastroenteritis. †Credible interval not available for all studies. ‡The study was limited to campylobacteriosis, salmonellosis, listeriosis, infection with Shiga toxin-producing *Escherichia coli*, yersiniosis, and infection with norovirus. §The reported/estimated cases of severe illness in this study can be considered to be approximately the same as hospitalizations.

Our finding that brucellosis, salmonellosis, echinococcosis, and toxoplasmosis together accounted for ≈70% of annual DALY means that these diseases might be major targets for policy making regarding appropriate food safety management actions, especially because their causative agents and likely transmission routes are generally known. Overall, the approach may be of interest to competent authorities in other countries requiring risk-based estimates ranking the impact of foodborne pathogens on public health to prioritize risk management actions.

## Supplementary Material

Technical Appendix Selection of input distributions and other parmameters for the disability-life year estimates.

## References

[1] Flint JA, van Duynhoven YT, Angulo JF, de Long MS, Braun P, Kirk M, Estimating the burden of acute gastroenteritis, foodborne disease, and pathogens commonly transmitted by food: an international review. Clin Infect Dis. 2005;41:698–704. 10.1086/432064 16080093

[2] World Health Organization. The global burden of foodborne disease: taking stock and charting the way forward: WHO consultation to develop a strategy to estimate the global burden of foodborne diseases, Geneva, September 25–27, 2006 [cited 2010 Mar 26]. http://www.who.int/foodsafety/publications/foodborne_disease/fbd_2006.pdf

[3] Mead PS, Slutsker L, Dietz V, McCaig FL, Breese SJ, Shapiro C, Food-related illness and death in the United States. Emerg Infect Dis. 1999;5:607–25. 10.3201/eid0505.990502 10511517 PMC2627714

[4] Rocourt J, Moy G, Vierk K, Schlundt J. The present state of foodborne disease in OECD countries. Geneva: World Health Organization; 2003 [cited 2010 Oct 20]. http://www.who.int/foodsafety/publications/foodborne_disease/en/OECD%20Final%20for%20WEB.pdf

[5] Havelaar AH, Galindo AV, Kurowicka D, Cooke RM. Attribution of foodborne pathogens using structured expert elicitation. Foodborne Pathog Dis. 2008;5:649–59. 10.1089/fpd.2008.0115 18687052

[6] Havelaar AH, van Duynhoven YT, Nauta MJ, Bouwknegt M, Heuvelink AE, De Wit GA, Disease burden in The Netherlands due to infections with Shiga toxin–producing *Escherichia coli* O157. Epidemiol Infect. 2004;132:467–84. 10.1017/S0950268804001979 15188716 PMC2870126

[7] National Institute for Public Health and the Environment. Our food our health. Healthy diet and safe food in the Netherlands. 2006. Report No 270555009 [cited 2010 Mar 26]. http://www.rivm.nl/bibliotheek/rapporten/270555009.pdf

[8] Melse JM, Essink-Bot ML, Kramers PG, Hoeymans N. A national burden of disease calculation: Dutch disability-adjusted life years. Am J Public Health. 2000;90:1241–7. 10.2105/AJPH.90.8.1241 10937004 PMC1446331

[9] Hellenic Statistical Authority. Pireaus: General Secretariat of the National Statistical Service of Greece [cited 2010 Mar 25]. http://www.statistics.gr

[10] Center for Infectious Diseases Control. Marousi: Ministry of Health and Welfare: Hellenic Center for Infectious Diseases Control [cited 2010 Mar 25]. http://www.keelpno.gr

[11] World Health Organization. WHO Surveillance Programme for Control of Foodborne Infections and Intoxications in Europe. 8th Report 1999–2000. Country Reports: Greece. 1999–2000 [cited 2010 Mar 26]. http://www.bfr.bund.de/internet/8threport/CRs/gre.pdf

[12] World Health Organization. WHO Surveillance Programme for Control of Foodborne Infections and Intoxications in Europe. 7th Report. Country Reports: Greece 1993–1998; 2003 [cited 2010 Mar 26]. http://www.bfr.bund.de/internet/7threport/CRs/GRE.pdf

[13] Denny J, McLaughlin J. Human *Listeria monocytogenes* infections in Europe—an opportunity for improved European surveillance. Euro Surveill. 2008;13:8082. 18445429

[14] Mossialos E, Allin S, Davaki K. Analysing the Greek health system: a tale of fragmentation and inertia. Health Econ. 2005;14:S151–68. 10.1002/hec.1033 16161195

[15] Diza E, Frantzidou F, Souliou E, Arvanitidou M, Gioula G, Antoniadis A. Seroprevalence of *Toxoplasma gondii* in northern Greece during the last 20 years. Clin Microbiol Infect. 2005;11:719–23. 10.1111/j.1469-0691.2005.01193.x 16104986

[16] Papazahariadou MG, Papadopoulos EG, Frydas SE, Mavrovouniotis C, Constantinidis TC, Antoniadou-Sotiriadou K, Prevalence of gastrointestinal parasites in the Greek population: local people and refugees. Annals of Gastroenterology. 2004;17:194–8.

[17] Hall G, Kirk DM, Becker N, Gregory EJ, Unicomb L, Millard G, Estimating foodborne gastroenteritis, Australia. Emerg Infect Dis. 2005;11:1257–64. 16102316 10.3201/eid1108.041367 PMC3320479

[18] Scallan E, Hoekstra RM, Angulo FJ, Tauxe RV, Widdowson MA, Roy SL, Foodborne illness acquired in the United States—major pathogens. Emerg Infect Dis. 2011;17:7–15. 21192848 10.3201/eid1701.P11101 PMC3375761

[19] McManus DP, Zhang W, Li J, Bartley PB. Echinococcosis. Lancet. 2003;362:1295–304. 10.1016/S0140-6736(03)14573-4 14575976

[20] Pappas G, Papadimitriou P, Akritidis N, Christou L, Tsianos EV. The new global map of human brucellosis. Lancet Infect Dis. 2006;6:91–9. 10.1016/S1473-3099(06)70382-6 16439329

[21] Vijgen SMC, Mangen MJJ, Kortbeek LM, van Duijnhoven YTHP, Havelaar AH. Disease burden and related costs of cryptosporidiosis and giardiasis in the Netherlands. Bilthoven: National Institute for Public Health and the Environment, 2007 [cited 2010 May 14]. http://www.rivm.nl/bibliotheek/rapporten/330081001.pdf

[22] World Health Organization [cited 2009 Dec 12]. http:// www.who.int

[23] Kemmeren JM, Mangen MJJ, van Duynhoven YTHP, Havelaar AH. Priority setting of foodborne pathogens: disease burden and costs of selected enteric pathogens. Bilthoven: National Institute for Public Health and the Environment; 2006. 330080001 [cited 2010 May 14]. http://www.rivm.nl/bibliotheek/rapporten/330080001.pdf

[24] Van Lier EA, Havelaar AH. Disease burden of infectious diseases in Europe: a pilot study. Bilthoven: National Institute for Public Health and the Environment. 2007. Report No 215011001 [cited 2010 Mar 26]. http://www.rivm.nl/bibliotheek/rapporten/215011001.pdf

[25] World Health Organization. Mortality Database [cited 2009 Mar 25]. http://www.who.int/whosis/mort/download/en/index.html

[26] McCabe-Sellers BJ, Beattie SE. Food safety: emerging trends in foodborne illness surveillance and prevention. J Am Diet Assoc. 2004;104:1708–17. 10.1016/j.jada.2004.08.028 15499359

[27] Vorou R, Dougas G, Gkolfinopoulou K, Mellou K. Gastroenteritis outbreaks in Greece. The Open Infectious Diseases Journal. 2009;3:99–105. 10.2174/1874279300903010099

[28] Ekdahl K, Giesecke J. Travellers returning to Sweden as sentinels for comparative disease incidence in other European countries, *Campylobacter* and *Giardia* infection as examples. Euro Surveill. 2004;9:6–9. 15381837

[29] Adak GK, Long SM, O'Brien SJ. Trends in indigenous foodborne disease and deaths, England and Wales: 1992 to 2000. Gut. 2002;51:832–41. 10.1136/gut.51.6.832 12427786 PMC1773489

[30] Vorou R, Gkolfinapoulou K, Dougas G, Mellou K, Pierroutsakos IN, Papadimitriou T. Local brucellosis outbreak on Thassos, Greece: a preliminary report. Euro Surveill. 2008;13:pii:18910. 18761941

[31] Minas M, Minas A, Gourgulianis K, Stournara A. Epidemiological and clinical aspects of human brucellosis in central Greece. Jpn J Infect Dis. 2007;60:362–6. 18032835

[32] Sotiraki S, Himonas C, Korkoliakou P. Hydatidosis-echinococcosis in Greece. Acta Trop. 2003;85:197–201. 10.1016/S0001-706X(02)00273-5 12606097

[33] Kardaras F, Kardara D, Tselikos D, Tsoukas A, Exadactylos N, Anagnostopoulou M, Fifteen year surveillance of echinococcal heart disease from a referral hospital in Greece. Eur Heart J. 1996;17:1265–70. 8869869 10.1093/oxfordjournals.eurheartj.a015045

[34] ECDC. The first European communicable disease epidemiological report. Stockholm: European Centre for Disease Prevention and Control; 2007 [cited 2099 Jul 28]. http://www.ecdc.europa.eu/en/publications/Publications/0706_SUR_First_%20Annual_Epidemiological_Report_2007.pdf

[35] World Health Organization/Food and Agriculture Organization of the United Nations. Risk assessments of *Salmonella* in eggs and broiler chickens. Geneva/Rome: The Organizations; 2002. ISSN 1726–5274 [cited 2009 Jul 29]. http://www.who.int/foodsafety/publications/micro/en/salmonella.pdf

[36] Havelaar AH, Kemmeren JM, Kortbeek LM. Disease burden of congenital toxoplasmosis. Clin Infect Dis. 2007;44:1467–74. 10.1086/517511 17479945

[37] de Jong B, Ekdahl K. The comparative burden of salmonellosis in the European Union member states, associated and candidate countries. BMC Public Health. 2006;6:4. 10.1186/1471-2458-6-4 16403230 PMC1352352

[38] Arnesen T, Kapiriri L. Can the value choices in DALYs influence global priority-setting? Health Policy. 2004;70:137–49. 10.1016/j.healthpol.2003.08.004 15364144

[39] Lake RJ, Cressey JP, Campbell MD, Oakley E. Risk ranking for foodborne microbial hazards in New Zealand: burden of disease estimates. Risk Anal. 2010;30:743–52. 10.1111/j.1539-6924.2009.01269.x 19645753

[40] Scallan E, Griffin PM, Angulo FJ, Tauxe RV, Hoekstra RM. Foodborne illness acquired in the United States—unspecified agents. Emerg Infect Dis. 2011;17:17–22. 10.3201/eid1701.P21101 PMC3204615 21192849

