# Supplementary material for: Risk-based Estimate of Effect of Foodborne Diseases on Public Health, Greece
Source: Emerg Infect Dis. 2011 Sep;17(9):1581–90. doi: 10.3201/eid1709.101766 (PMC3322063; doi:10.3201/eid1709.101766)
Supplement: Technical Appendix — Selection of input distributions and other parmameters for the disability-life year estimates. [file 10-1766-Techapp_38p.pdf]

Article DOI: http://dx.doi.org/10.3201/eid1709.101766

Risk-based Estimate of Effect of Foodborne Diseases on Public Health, Greece

Technical Appendix

SELECTION OF INPUT DISTRIBUTIONS AND OTHER PARAMETERS FOR THE DISABILITY-ADJUSTED LIFE YEAR ESTIMATES

Reported cases: Reported cases of illnesses that may be transmitted through food were for the larger part collected from the Hellenic Statistical Authority (ELSTAT) (*1*) and the Hellenic Center for Infectious Diseases Control (HCIDC) (*2*). A limited number of data were obtained from WHO disease surveillance reports where HCIDC was mentioned to be the source (*3,4*) for better transparency and from other literature when no other information was available (*5*). The study included the period 1996 through 2006 for which data were available from both national sources (Technical Appendix Technical Appendix Table 1). ELSTAT collects information regarding hospitalizations for cases that have a duration of stay of at least 1 day based on the Basic Tabulation List (BTL) of the International Classification of Diseases, Ninth Revision (ICD-9). ELSTAT data are based on sampling of hospitalized patients' bulletins. This sampling includes bulletins of deceased patients though the latter are not recorded separately. Recorded hospitalizations by the ELSTAT are likely to differ in their severity as Greece's population had free access to hospital centers during the period of the study where it was possible to be treated even for minor health complaints (*6*). HCIDC collects information on notified cases from hospital microbiological laboratories and district health authorities (*3*) while it also performs active surveillance on the general incidence of gastroenteritis in the country through physicians (*2*). HCIDC data can thus be representative of hospitalizations or visits to physicians and they are a mixture of laboratory confirmed and symptom based notified cases. For the few gastrointestinal illnesses (shigellosis, typhoid and paratyphoid fever) for which data were available from both sources, cases were usually more for hospitalizations (ELSTAT data) than for notified cases (HCIDC data). Among the non-gastrointestinal illnesses (echinococcosis and brucellosis) this was always the case. This could be due to the fact that either there is serious

undernotification (both by hospitals and physicians) to the HCIDC or extended repetition of tests and prescriptions in hospitals as suggested by Mossialos et al. (*6*) that could result in more than one bulletin for the same person. For the purpose of our estimates, we considered both ELSTAT and HCIDC data to be representative of reported (severe) cases of illness and we decided not to include undernotification (or possible overnotification) because a correction for these phenomena would require a country specific study on the validation of the 2 systems, which is not available. For the four illnesses for which data were available from both systems, ELSTAT data were preferred because they are based on sampling of bulletins directly from hospitals while part of the HCIDC data are collected indirectly through district health authorities (*3*).

Percentage foodborne: To cover for the uncertainty associated with food attribution, program evaluation and review technique (PERT) distributions were used as multipliers (*7*). The minimum and maximum parameters of the PERT distributions were based on a literature search covering the range of potential values for this factor for every illness, and the most likely values were based on data most relevant to Greece/Europe as the endemicity of illnesses is often related to a specific region (*8–10*). When no European data could be found, data from other industrialized countries (*7,11*) and in the absence of the latter expert elicitation data (*12*) were used. When no other information was available to construct an upper limit for foodborne transmission a value of 100% was assumed as in the case of brucellosis, echinococciasis, amoebiasis and other helminthiases. A synopsis of the selected parameters accompanied by the country of origin of the data are provided in Technical Appendix Technical Appendix Table 2. More detailed information about the range of values found for every illness during the literature search is provided below.

Underreporting: To cover for the uncertainty associated with under-reporting, Pert distributions were used as multipliers (*7*). The minimum and maximum parameters of these distributions were selected to cover the full range of values found in literature for this factor and the most likely values were set at the middle of this range for every illness since unlike food attribution there is no particular reason to consider European data more representative than data from other industrialized countries for this phenomenon. A synopsis of the selected parameters accompanied by the country of origin of the data are provided in Technical Appendix Technical Appendix Table 2. More detailed information about the range of values found for every illness during the literature search is provided below.

Case-fatality rate: To cover for the uncertainty associated with the case-fatality ratio, PERT distributions were also used as multipliers. The minimum and maximum parameters of these distributions were selected so as to cover the range of values found in literature for this factor and the most likely values were set at the middle of this range for every illness. The literature search was focused on reviews for specific pathogens, outbreak studies and national burden of disease studies. The selected case fatality ratios from these studies were always based on reported cases of serious illness resulting in physician visits so that they are on the same level of the surveillance pyramid as the cases reported in Greece's surveillance system. In some instances few national data were available to estimate case fatality rates for generic codes ("Ill-defined intestinal infections," "Other helminthiases" and "Intestinal infections due to other specified microorganism") found in the WHO Mortality Database (*13*). A synopsis of the selected parameters accompanied by the country of origin of the data are provided in Technical Appendix Technical Appendix Table 2. More detailed information about the range of values found for every illness during the literature search is provided below.

Disability weights: To select the disability weights, studies were collected from literature in which the burden of every illness was quantified by using disability-adjusted life years (DALYs). Selected studies involved review studies on the global burden of specific illnesses (e.g., echinococcosis [*14*], brucellosis [*15*]) or on the burden of foodborne pathogens in the European Union (EU) or the Netherlands and other countries where the same indicator was used (*16–19*). For generic codes involving gastrointestinal illness due to various causes (e.g., "Other helminthiases") or unknown causes (e.g., "Ill-defined intestinal infections") disability weights were selected conservatively by using the classification system by Murray (*20*) in such a way that they would coincide with the upper range of every disability weight class. For example in the case of the ill-defined illness a disability weight of 0.4 was selected which corresponds to the upper range of class 3 disability weights (ranging from 0.220–0.4) used to describe severe gastrointestinal illness in other studies (e.g., 0.393 for severe gastroenteritis due to enterohemorrhagic *Escherichia coli* (EHEC) and giardiasis [*16,18*]). For underreported mild cases of gastrointestinal illness when no data on disability weights could be found in literature (e.g., "Ill-defined intestinal infections" or "Amebiasis") given the similarity of mild diarrheal symptoms of infectious origin (*21*) a disability weight of 0.067 was chosen as in other studies

where weights are assigned to mild diarrheal symptoms (*18,22*). A synopsis of all the selected disability weights is provided in Technical Appendix Technical Appendix Table 3.

SOFTWARE

We performed all estimations using the @RISK 5.7 software (Palisade Corporation, Ithaca, NY, USA) as an add-in in Microsoft Excel 2010 (Microsoft, Redmond, WA, USA) based on one simulation of 100,000 iterations, performed by applying the Latin Hypecube technique using a fixed seed value of 1 to eanable our results to be reproduced by others and to make possible comparisons between alternative inputs.

BACTERIAL DISEASES

Botulism

Reported cases: Based on HCIDC data (Technical Appendix Technical Appendix Table 1).

Percentage foodborne: According to Abgueguen et al., 80%–100% of cases of botulism in the EU are foodborne (*23*) and 100% of the cases in France have been attributed to food (*24*). Given the fact that wound botulism, which is the only form of the disease not related with food, occurs very rarely in Europe (*25*) the estimate of 100% was chosen for Greece.

Case-fatality rate: The most likely case-fatality rate was considered to be 10.15%, the average of the range suggested by US data (3%–17.3%) (*7,26*).

Disability weight: It is estimated that ≈25% of the patients have to be intubated (severe cases) (*26*) and the remaining 75% are considered to be moderate cases. Disability weights of 0.906 were assigned to the severe cases and 0.6 to the moderate cases based on the description of the symptoms of the latter by Mann et al. (*27*) and the definitions of disability weights by Murray (*20*).

Duration of illness: The duration of illness was estimated to be 60 days for the patients ending up in need of mechanical help for respiration according to EU studies (*23,28*) while for the moderate cases the reported hospitalization time is 1 (*27*) to 2 weeks (*29*). The latter value was considered to be more reliable since it is based on estimates from a much larger outbreak of botulism that did not involve any pending legal actions unlike the former value.

Underreporting: A factor of 1.8125 was considered which is in the middle of the range 1.625–2 (*11,25*).

Note: The reported cases for Greece during 1998–2007 do not involve any incidents of infant botulism (*2*). Reported and underreported cases were considered to be equally severe. Although mild cases of botulism can also occur and they have often been reported during outbreaks, they were not considered here because affected persons seldom seek medical care and are not normally captured by surveillance unless they are part of an outbreak (*7*). Annually reported cases in Greece are ≤1 and they do not fit the outbreak definition (≥2 cases) (*30*).

Brucellosis

Reported cases: Based on ELSTAT data (ICD-9 code 023; BTL code 031) (Technical Appendix Technical Appendix Table 1).

Percentage foodborne: In a Greek study foodborne transmission was mentioned to be 84% (*31*) and in US studies 50% (*7,11*). Given the fact that brucellosis is a disease that is considered to have a high transmission rate through food (*32*) the value from the Greek study was selected to represent the mean of the PERT distribution. The minimum value of the distribution was based on the US studies, and the maximum value on an arbitrarily assigned conservative value of 100% since no other upper limit has been found in literature.

Case-fatality rate: The case-fatality rateof brucellosis is 5% based on Mead et al. (*11*) but on the HCIDC Web site it is stated that the mortality rate for the disease is less than 2% (*2*) and Roth et al. mention that the mortality rate is negligible although the disease can last for several years (*15*). A case-fatality rate of 2% was finally selected since it was considered to be closer to the situation in Greece (*33*). We considered 0.9% as a lower limit for the case-fatality rate based on recent US data (*7*).

Disability weight: A disability weight of 0.2 was assigned as in the study by Roth et al. based on the fact that the disease is perceived as painful and affecting occupational ability even during periods of remission (*15*).

Duration of illness: The median duration of the disease (clinical manifestation) is 3.11 years based on Roth et al. (*15*).

Underreporting: Given the seriousness of the disease whose clinical manifestations can last for years the underreporting factor would in theory be expected to be low (e.g., 2) as suggested for serious illnesses (*11*). Nonetheless, it can be as high as 19.7 based on estimates for the annual incidence of the disease in certain regions in Greece (*34*) and the average annual incidence of hospitalizations due to brucellosis during 1996–2005 (*1*). The average of these extremes was selected (10.85).

Note: Reported and underreported cases were considered to be equally severe.

Campylobacteriosis

Reported cases: Based on HCIDC data (Technical Appendix Technical Appendix Table 1).

Percentage foodborne: The percentage of cases of campylobacteriosis that can be attributed to food ranges from 30% to 80% based on various European studies summarized by Havelaar et al. (*12*). Although some of these studies mention 80% of the cases to be foodborne (*11,35*) data from a recent publication of the European Food Safety Authority on campylobacteriosis in the EU point out toward lower food attribution values, since only 35% of outbreaks and less than 50% of outbreak cases based on this study are due to food and drinking water (*36*) and the same is the case for another recent expert elicitation study according to which only 42% of the cases are due to food (*12*). Therefore, given this large variation among studies the average of the reported range of food attribution values in the European studies was selected as the most likely value for the PERT distribution (55%).

Case-fatality rate: A case fatality rate of 0.1265% was selected, which is in the middle of the observed range 0.1–0.153 for reported cases (*7,11,35*).

Disability weight: Different disability weights were assigned to each of the potential outcomes for reported cases of campylobacteriosis based on van Lier and Havelaar (*16*) and Haagsma et al. (*37*) as can be seen in Technical Appendix Technical Appendix Table 4. All underreported cases were assumed to be cases of self-limiting gastroenteritis and were thus assigned a disability weight of 0.067 (*38*).

Duration of illness: The estimated duration of each outcome for the reported cases can be seen in Technical Appendix Technical Appendix Table 4. The underreported cases were

assumed to have a duration of 3.48 days, which corresponds to gastroenteritis due to *Campylobacter* that does not result in a visit to GP (*22*).

Underreporting: Mead et al. mention that the disease is under-reported by a factor of 38 (*11*). According to European studies under-reporting of campylobacteriosis is estimated to be much lower; 7.6 based on Wheeler et al. (*39*) and 10.3 based on Adak et al. (*35*). However, taking into account that in Greece very few laboratories have the ability to identify the pathogen (*2*) the underreporting is expected to be higher than in other European countries and this was indeed found to be the case in a study by Ekdahl and Giesecke (*40*). However, in the latter study the incidence data for campylobacteriosis in Greece used to derive the underreporting factor where much lower than what is known for the year 2000 based on WHO data. Therefore by repeating the calculations for the correct national data the under-reporting factor was estimated as:

$$\frac{\left(\dfrac{\text{risk per 100,000 inhabitants in Finland}}{\text{risk per 100,000 travellers returning from Finland}}\right)}{\left(\dfrac{\text{risk per 100,000 inhabitants in Greece}}{\text{risk per 100,000 travellers returning from Greece}}\right)} = \frac{\left(\dfrac{68.2}{0.9}\right)}{\left(\dfrac{2.473}{17.7}\right)} = 542$$

To cover for uncertainty due to this parameter the whole range of under-reporting factors was considered (7.6–542) and the most likely value (274.8) was considered to be at the middle of this range.

EHEC

Reported cases: Based on HCIDC data (Technical Appendix Technical Appendix Table 1).

Percentage foodborne: Approximately 51% of the sources of EHEC outbreaks are foodborne or waterborne in Europe according to a study by Ammon for 1992–1996 (*41*). The range of values for this percentage could be larger (40%–90%) (*12*).

Case-fatality rate: The case-fatality rate for *E. coli* O157 has been estimated to be in the middle of the range suggested for EHEC O157 (0.25%–0.83%) (*11,42*).

Disability weight: There are four major conditions related with EHEC infection that determine the burden of the disease, watery diarrhea, hemorrhagic colitis, hemolytic-ureamic

syndrome (HUS) (*43*) and end-stage renal disease (ESRD). For watery diarrhea and hemorrhagic colitis a disability weight of 0.393 was assigned based on van Lier and Havelaar (*16*). It was also considered that for every reported case of EHEC, 0.5 cases of HUS occur, and that every case of HUS (including ESRD as a sequela) corresponds to 1.05 YLD (*16*). All underreported cases were considered to be cases of mild gastroenteritis for which a disability weight of 0.067 was selected (*38*).

Duration of illness: The duration of EHEC-related gastroenteritis varies between 2–9 days (*44*). Here, an average duration of 5.6 days was selected for watery diarrhea and hemorrhagic colitis due to EHEC infection according to van Lier and Havelaar (*16*). For the under-reported cases a duration of 3 days was assumed based on the median duration of non-bloody shiga toxigenic *Escherichia Coli* (STEC) O157 gastroenteritis (*22*).

Underreporting: An underreporting factor of 2 has been mentioned for verocytotoxin producing Escherichia coli (VTEC) in England and Wales (*35*) and 26.1 has been mentioned for the United States. (*7*). The most likely value (14.05) was considered to be in the middle of this range. Given the fact that cases of EHEC have only recently started to be under surveillance in Greece (*2*) undernotification of EHEC is also possible.

Leptospirosis

Reported cases: Based on HCIDC data (Technical Appendix Technical Appendix Table 1).

Percentage foodborne: Leptospirosis is contracted by direct or indirect contact with the urine of an infected animal (*45*) and thus common vehicles of the infection are contaminated food, water and soil (*46*). Though leptospirosis is considered to be a foodborne disease (*46,47*) estimating an actual percentage of cases that can be attributed to food is complicated since this disease has various ways of transmission (*46*). In a recent review of leptospirosis in the north of Greece, the source of the infection had been identified in only 51% of the cases studied where it was found to be non-food related (*48*). This means that the percentage of cases that can be attributed to food is anywhere between 1 and 49%. Taking into account another review of the disease in Germany where different types of exposure are mentioned (*49*) it can be concluded that food is not a common vehicle of the disease but keeping in mind that the only outbreak of leptospirosis in Greece reported in literature was foodborne (involved drinking water at a cafe)

(*45*) we assumed a very low food attribution of 5% which is in the same range as the one of hepatitis A or *Rotavirus* infections (*12*).

Case-fatality rate: The severity of the disease caused by leptospirosis can vary considerably, ranging from a subclinical infection to a severe syndrome of multi-organ infection with high mortality (*45*). Among those that become ill, leptospirosis has two clinically recognizable syndromes, the anicteric form (90% of patients) and the icteric form or Weil syndrome (*50*). Deaths from the anicteric form of the disease have been reported as rare (*50*) or almost nil (*45*) and in the ≈10% of the patients that have the icteric form there is an associated 10% mortality rate (ranging from 5% to 10%) (*45,50*). In our study we assumed all reported cases to be representative of the icteric form of the disease and thus the latter case fatality rate was considered.

Disability weight: The anicteric form includes the great majority of infections which are either subclinical or of very mild severity for which we assumed a class 1 disability weight of 0.096 (*20*). The icteric form of the disease is very severe and is characterized by impaired renal and hepatic function, hemorrhage, vascular collapse, and mental status changes (*50*) and can have severe sequelae like acute renal failure, which occurs in 16 to 40% of the cases (*45*). Thus, for icteric leptospirosis a class 6 disability weight of 0.920 was assumed (*20*).

Duration of illness: Leptospirosis is listed in the ICD 10 coding system under code A27. Based on the average of countries that have submitted information in the EHMD for this disease in the years 2004–2006 we have considered the average duration of hospitalization to be ≈12 days (*51*). The underreported cases are assumed to belong to people that present the anicteric form of the disease which is not likely to result in medical consultation and lasts for about a week (*45*).

Underreporting: We assumed that all reported cases belong to the icteric form of the disease which is serious and that all underreported cases can be attributed to the anicteric form of the disease. Thus, an under-reporting factor of 15 was applied since the ratio of icteric/anicteric leptospirosis has a possible range between 1/20 and 1/10 (*45*).

Listeriosis

Reported cases: Based on HCIDC data. For 2001 and 2002 data from a European report on listeriosis in EU countries (*5*) were used since no data were available from the HCIDC for these years (Technical Appendix Table 1).

Percentage foodborne: 99% of the cases were considered to be foodborne (*35*). Other authors, however, mention this percentage being in the range of 69%–99% (*12*) or 100% (*7*).

Case-fatality rate: The case-fatality rate can range between 10% and 44% based on outbreak data covering both perinatal and other outbreak cases of severe listeriosis tabulated by Schlech (*52*). The average of these extreme values was considered (27%)

Disability weight: No disability weight was assigned to this disease since the burden of illness is determined mainly by the fatal cases (*22*). Moreover given the variety of symptoms related with either postnatal or adult acquired listeriosis (meningitis, sepsis, septicemia, pneumonia, abortion to mention but a few) assigning a uniform disability weight is extremely complex.

Duration of illness: For the same reason, as mentioned for disability weight, no assumptions were made regarding the duration of illness.

Underreporting: An underreporting factor of 1.7 was selected, which is in the midlle of the range found in literature for this pathogen 1.1–2.3 (*7,53*) Under-reported cases were assumed to have the same severity as reported cases. Listeriosis is also the only disease in our study for which underreported cases were also used in estimating the total number of deaths. The reason for this is that the disease has only recently started to be under surveillance in Greece by the HCIDC and it is thus expected to be under-notified. Given again the fact that the DALY due to listeriosis are defined by the fatal cases any under-notification of the disease is likely to lead to a considerable underestimation of its true impact on the health of a population. Thus deaths are also estimated based on the expected number of underreported cases.

Note: mild cases of listeriosis resulting in diarrhea are not considered in this study.

Salmonellosis

Reported cases: Based on HCIDC data (Technical Appendix Table 1).

Percentage foodborne: 95% of the cases are considered to be foodborne (*11,24*). However, this value may also be considered to be 55%–95% (*12*).

Case fatality: The case fatality rate is considered to be 0.701% which is in the middle of the range 0.5%–0.902% suggested for laboratory confirmed cases (*7,35*).

Disability weight: According to other studies on the burden of illness (*16,37*) salmonellosis has 4 potential outcomes: gastroenteritis, inflammatory bowel disease, irritable bowel syndrome and reactive arthritis for which the incidence and disability weights are presented in Technical Appendix Table 5. For the underreported cases, which we assumed that they did not result in a visit to a physician, a disability weight of 0.067 was selected (*22*).

Duration of illness: The duration of illness for reported cases of illness is shown in Technical Appendix Table 5. For the under-reported cases a duration of 5.58 days was assumed based on the median duration of gastroenteritis in salmonellosis patients that do not visit a GP (*22*).

Under-reporting: Mead et al. mention that the disease is under-reported by a factor of 38 (*11*) and in European studies underreporting factors of 3.2 (*39*) or 3.9 (*35*) are mentioned though in a recent study by de Jong and Ekdahl the underreporting factor for several other European countries was mentioned to be much higher and in particular for Greece 99.7 (*54*). Thus the whole range was considered (3.2–99.7) with a most likely value set at the middle of the extreme values (51.45).

Shigellosis

Reported cases: Based on HCIDC and ELSTAT data (ICD-9 code 004; BTL code 012) (Technical Appendix Table 1). HCIDC data were not available for 1996–2003 and then ELSTAT data were used. For the remainder years (2004–2006) data from the HCIDC were used.

Percentage foodborne: 10% of the cases were considered as foodborne (*24*). This value may nonetheless range between 8.2 based on data from England (*35*) and 31% based on recent US data (*7*).

Case-fatality rate: The case-fatality rate was considered to be 0.1%–0.16% (*7,11*) with the average (0.13%) as the most likely value.

Disability weight: A disability weight of 0.22 was selected based on Wijewardene and Spohr (*19*). For the under-reported cases a disability weight of 0.067 was selected (*22*). To describe sequelae related with irritable bowel syndrome the same disability weight as for campylobacteriosis and salmonellosis derived IBS was used (*37*).

Duration of illness: The duration of hospitalizations (4.6 days) is based on data from the ELSTAT. For the under-reported cases the duration of illness was assumed to be the same as for *E. coli* O157 i.e., 3 days (*22*). For irritable bowel syndrome the same duration as for campylobacteriosis and salmonellosis derived IBS was used (*37*).

Under-reporting: Scallan et al. mention an under-reporting factor of 33.3 (*7*) and Adak et al. report a factor of 3.4 (*35*). The whole range was considered (3.4–33.3) with a most likely value set at the middle of the extreme values (18.35).

Typhoid and paratyphoid fever

The clinical manifestation of paratyphoid fever is very similar to the one of typhoid fever though usually milder (*55*). Nonetheless, given the fact that these infections are discussed together we also decided to treat them as one when estimating their health burden.

Reported cases: Based on HCIDC and ELSTAT data (ICD-9 code 002; BTL code 011) (Technical Appendix Table 1).

Percentage foodborne:. 80% of the total cases are estimated to be foodborne based on data from England and Whales (*35*). Nonetheless, variation could be much greater if we accept the range of what is considered foodborne for *Salmonella* spp. (55%–95%) (*12*).

Case fatality: Is in the range of 0.4%–1.5%. The average of these extremes (0.95%) was considered to be the most likely value (*11,24*).

Disability weight: A disability weight of 0.096 has been selected by Wijewardene et al. (*19*). However, considering that the disability weight for non typhoidal gastroenteritis due to salmonellosis is 0.393 only for hospitalized cases according to van Lier and Havelaar (*16*) a disability weight of 0.096 does not reflect the severity of the disease. Therefore it was decided to arbitrarily assign a class 4 disability weight of 0.6 to severe cases of typhoid fever (hospitalizations) and a class 1 disability weight of 0.096 to uncomplicated cases that can be

treated at home since no other studies regarding the severity weight of this disease have been found in literature (*20*).

Duration of illness: Acute cases were assumed to last ≈2 weeks and uncomplicated cases usually 1 week (*55,56*).

Under-reporting: An under-reporting factor of 2 has been used for typhoid fever (*11, 35*) but a factor of 13.3 has also been suggested (*7*). The most likely value was set at the middle of this range (7.65).

Food poisoning

Reported cases: Based on ELSTAT data (ICD-9 codes 003, 005; BTL code 013) (Technical Appendix Table 1).

The BTL code under this item includes the following two ICD-9 codes (*57*):

Other salmonella infections (003) and

Other food poisoning (bacterial) (005). The latter category includes:

- Staphylococcal food poisoning
- Botulism
- *Cl. perfringens* food poisoning
- Food poisoning due to other *Clostridium* species
- Food poisoning due to *Vibrio parahemolyticus*
- Other microbial food poisoning (*B. cereus* food poisoning)
- Other unspecified food poisoning

Since we have separate data for the category "other salmonella infections" and "botulism" though the HCIDC, these have been used separately to estimate disease burden due to salmonellosis and botulism. Given the fact that fish and shellfish are always consumed cooked in Greece *V. parahemolyticus* is not expected to be a pathogen of concern to the public health although it has been isolated from fresh fish in the country (*58*) and is currently not included in the microorganisms that are notified to the HCIDC. *Bacillus cereus*, *Staphylococcus aureus*, and

Clostridium perfringens are mentioned as the most frequent causes of food poisoning in different countries (*59–63*) and thus it was assumed that the DALY due to food poisoning are defined by these three microorganisms. The short duration of hospitalization in Greece for food poisoning (weighted average of 4 days based on data from 1998–2005 that include cases of salmonellosis and botulism that are known to last longer) supports this assumption.

Percentage foodborne: By definition 100% is foodborne. Nonetheless, in some studies the percentage foodborne of the diseases caused by *S. aureus*, *B. cereus* and *C. perfringens* is somewhat lower ranging from 87%–91% (*12*).

Case fatality: Food poisoning due to the above microorganisms is generally self-limiting with an extremely low case fatality ratio, that ranges from 0 to 0.05% for these 3 pathogens (*11,64*). The average of this range (0.025%) was considered as the most likely value.

Disability weight: *B. cereus*, *St. aureus* and *Cl. perfringens* cause mild, self-limiting illnesses (*65*) so a class 1 disability weight of 0.067 was assigned to under-reported cases to match the disability weights of other underreported gastrointestinal illnesses included in this study and a class 2 disability weight of 0.220 to reported cases based on Murray, 1994 (*20*).

Duration of illness: Recovery from this kind of food poisoning is rapid and ranges usually from 1 to 3 days (*44,66*). The highest observed duration of illness (3 days) was selected for hospitalized cases and the lowest 1 day for under-reported cases.

Under-reporting: An under-reporting factor was difficult to establish since we do not know the relative incidence of *B. cereus*, *S. aureus* and *C. perfringens* food poisoning cases in this item. Nonetheless, since *S. aureus* has been mentioned to be responsible for more than half of documented food poisoning cases (*59*) an under-reporting factor of 185.65 was selected which is in the middle of the range found for this pathogen 29.3–342 (*7,53*).

PARASITIC DISEASES

Amebiasis

Reported cases: Based on ELSTAT data (ICD-9 code 006; BTL code 014) (Technical Appendix Table 1).

Percentage foodborne: Infection is initiated by ingestion of faecally contaminated food or water (*43*) and water, food and food-handlers have been reported to be the sources of infections (*67*) so in theory 100% of cases can be foodborne. However, the cysts of *Entamoeba histolytica* can survive in water (*68*) meaning that swimming in pools and beaches can also result in infections (*69*) and venereal transmission has also been observed (*70*). Moreover, the role of insects in the transmission of the disease has not been properly investigated (*71*). Given these uncertainties we assumed that 50% of the cases are due to food. In theory this percentage could be as low as 10% which was the case for foodborne transmission of *Giardia lamblia* which like *E. histolytica* is also transmitted by water, food and food handlers (*67*) but has been reported to be more often transmitted by faecally contaminated water than food (*72*).

Case fatality: The case fatality rate was considered to be ≈0.2% based on tabulated data by Walsh (*21*) regarding the global prevalence and incidence of amoebiasis (range: 0.1%–0.3%).

Disability weight: A value of 0.4 was selected based on Wijewardene et al. (*19*). For the cases that go under-reported a disability weight of 0.067 was selected (*22*) based on similarity of the mild diarrheal syndrome of amoebiasis with salmonellosis, giardiasis, toxigenic *E. coli* diarrhea, many other diarrheas of infectious origin, or the irritable bowel syndrome (*21*).

Duration of illness: Based on ELSTAT data the average duration of the illness was assumed to be 4.74 days based on the average duration of hospitalization according to the ELSTAT (*1*). For the under-reported cases the duration of diarrhea was assumed to be 3 days based on the same rationale of the disability weight selection and the fact that non-bloody diarrhea by STEC O157 lasts for 3 days (*22*).

Under-reporting: An under-reporting factor of 10 is plausible as suggested for protozoan infections by Casemore (*73*). According to Evangelopoulos et al, 2001 (*74*) the prevalence of *E. histolytica* in Greece is 1 in every 38 immigrants. Considering that the total population of immigrants (people of foreign nationality) in Greece in 2001 was 796,713 based on the ELSTAT (*1*), infected persons were 20,966. Approximately 10% of infected persons have symptoms, and out of these persons 2%–20% (average 11%) have symptoms related with the invasion of *E.*

histolytica beyond the intestinal mucosa (*68*) which are presumed to require hospitalization. Therefore ≈230 persons with amebiasis in 2001 are presumed to have required hospitalization. Nevertheless in 2001 only 25 people sought medical care (*1*) which means that the underreporting factor is at least 9.2. However, this is a conservative estimate based on the prevalence of the pathogen in only one (although the largest) of the high risk groups in developed countries (immigrants, travelers, migrant workers, immunocompromised persons, institutionalized persons, and sexually active homosexuals) (*68*). Here a factor of 9.6 was selected which is in the middle of the range (9.2–10).

Cryptosporidiosis

Estimated cases: No data are available for cryptosporidiosis so the number of cases is estimated indirectly as follows. The prevalence of *Cryptosporidium parvum* infection in the Greek population is 2.7% based on data collected from Papazahariadou et al. (*75*) which is in line with the available information on the incidence of this parasitic infection in Europe (*68*). We assumed that this rate of infection applies to all patients getting hospitalized for gastroenteritis where a protozoan infection could be the cause according to ELSTAT data (BTL Code 015, “intestinal infections due to other specified microorganism” and BTL code 016, “Ill-defined intestinal infections).

Percentage foodborne: Based on the study for the United States (*11*) 10% of the cases are foodborne while based on a study for England and Wales (*35*) 5.6% of the cases are attributed to food. The data resulting from the European study were considered to be more relevant for Greece.

Case-fatality rate: A range of 0.07%–0.6% has been mentioned (*18,35*) and the average has been considered to be the most likely value.

Disability weight: A disability weight of 0.393 was selected since in our scenario we estimated the cases of the disease as a proportion of the cases that result in visits to a physician for gastroenteritis or hospitalization. A disability weight of 0.067 was selected for the under-reported cases (*18*).

Duration of illness: The average duration of illness was considered to be 18.4 days for cases resulting in hospitalization and 3.5 days for cases not resulting in a visit to a physician (*18*).

Underreporting: The underreporting factors for *Cryptosporidium parvum* infections range from 7.4 (*35*) to 98.6 (*7*). We set the most likely value for under-reporting at the middle of this range.

Echinococcosis

It is assumed that all cases in Greece are due to *Echinococcus granulosus* which causes cystic echinococcosis that is easier to treat and has a lower case fatality than alveolar echinococcosis caused by *E. multilocularis* (*76*). This assumption is based on the fact that alveolar echinococcosis is a rare form of echinococcosis in Greece since this country is not among the endemic regions of *E. multilocularis* (*77*) and only one case has been reported between 1981 and 2000 (*78*). Moreover, according to a recent review on echinococcosis in Greece (*79*) it is rather certain that the majority if not all of the cases of the disease in humans in the country are due to *E. granulosus*. Additionally it is assumed that all cases of cystic echinococcosis are treated with surgery which still remains the only potentially curative treatment for cystic echinococcosis (*80*).

Reported cases: Based on ELSTAT data (ICD-9 code 122; BTL code 073) (Technical Appendix Table 1). Though HCIDC data were also available for the same period, ELSTAT data were used instead as they were considered of better quality. This was because the officially reported cases (through the HCIDC) have been found to be few in comparison to the actual number of diagnosed cases in hospitals in the country as mentioned by Sotiraki et al. (*79*).

Percentage foodborne: Cystic echinococcosis requires ingestion of the eggs (*76*) which can be the result of handling hosts of the parasite or ingesting food contaminated with eggs (*9*). The exact percentage of cases that can be attributed to food is not known (*32*). According to WHO (*81*), as much as 30% of cystic echinococcosis is transmitted through food by contamination with parasite eggs. Given the fact that the disease is hyperendemic in Greece (*9*) contamination of food from the environment can be high, so in our study 30% of the cases were considered to be foodborne. In theory though since *E. granulosus* is a helminth, foodborne transmission could be as high as 100% (see disease category “other helminthiases”).

Case-fatality rate: A case fatality rate of 2.24% was selected that corresponds to all cases receiving surgery in Greece and the neighboring countries (*14*). A wider range of 1%–3% is also possible (*76,82*) and was considered in the form of a Pert distribution.

Disability weight: Several disability weights were selected depending on the outcome of surgery for cystic echinococcosis according to Budke et al. (*14*) and are presented in Technical Appendix Table 6.

Duration of illness: The selected values are based on the recovery time reported for each possible outcome of surgery for cystic echinococcosis (*14*) and are shown in Technical Appendix Table 6.

Underreporting: Budke et al. (*14*) suggest an underreporting factor of 4 on a global level, this was not found to be very realistic for a western developed country given the seriousness of the disease. Moreover, echinococcosis is considered to be a serious health problem in Greece and the notification of the disease is obligatory in both humans and animals. An under-reporting factor of 2 based on the rationale of Mead et al. (*11*) for serious diseases is also possible. Given the fact that the reliability of official data was questioned in a recent review of the disease in the country (*79*) it was finally decided to select a factor of 3 which is in the middle of the above range. It was also assumed that 10% of the total cases are undiagnosed and thus do not receive medical treatment. These cases were assigned a disability weight of 0.2 for 10 years (*14*). Lastly, reported and under-reported cases were assumed to have the same severity.

Giardiasis

Estimated cases: No data are available for giardiasis so the number of cases is estimated indirectly as follows. The prevalence of the parasite in the Greek population according to a recent study is 2.3% (*75*) which is in line with what has been mentioned about the rates of detection of this parasite in industrialized countries (*68*). We assumed that this rate of infection applies to all patients getting hospitalized for gastroenteritis where a protozoan infection could be the cause (BTL Code 015, "intestinal infections due to other specified microorganism" and BTL code 016, "Ill-defined intestinal infections").

Percentage foodborne: Mentioned anywhere between 5 and 30% (*12*). Here a value of 10% was assumed as in (*35*).

Case fatality: The case fatality rate is very low according to Mead et al. (*11*) or zero according to Vijgen et al. (*18*) and this seems also to be the case in a more recent study by Adak et al. (*35*). However based on more recent U.S. data it can be 0.1% for laboratory confirmed cases (*7*). Thus a most likely value of 0.05% was considered which is in the middle of this range (0%–0.1%).

Disability weight: A disability weight of 0.393 was selected for hospitalized cases and 0.067 for under-reported cases (*18*).

Duration of illness: It was considered to be 10 days for people not visiting a physician and 30 days for hospitalized persons (*18*).

Under-reporting: This factor is in the range of 4.6–46.3 (*7,35*) and the average has been considered to be the most likely value.

Toxoplasmosis

Estimated cases: The disease has only recently started to be reported to the HCIDC (since 2004) and almost no cases were notified in the period 2004–2008 for which information is available (*2*). At the same time there are hospital studies on congenital toxoplasmosis that point out toward a much higher incidence of the disease in the country (*83,84*). Given this situation, it was considered more logical to estimate the total number of cases by multiplying the number of pregnancies for each year of the study with the percentage of seronegative women of reproductive age (~70%), the incidence of primary infection among pregnant women (0.51%) and the maternofetal transmission rate (19.4%) (*83,85,86*) (see Technical Appendix Table 7 of the Annex). The number of cases found in this way is in agreement with what is known so far regarding the incidence of the disease in developed countries which ranges between 1 and 10 cases per 10,000 births (*17*).

Percentage foodborne: In a European study by Cook et al. foodborne transmission for toxoplasmosis has been mentioned to be in the range of 30 to 63% (*87*). Here 50% of the cases were assumed to be foodborne since this value is approximately in the middle of the above range and has been used in other national studies as well (*7,11,24*).

Case fatality: The case-fatality rate is 2.3% according to Gibbs based on the percentage of stillbirths or neonatal deaths observed when toxoplasmosis occurs during pregnancy (*88*)

while according to Havelaar et al. (*17*) the incidence of fetal loss or neonatal death is 3.75% (3.3%–4.8%) among cases of congenital toxoplasmosis in the Netherlands. The case fatality rates by Havelaar et al. were selected for this study because they are more recent and derived through traceable and better quality data.

Disability weight: Toxoplasmosis can have various outcomes (fetal loss, neonatal death, chorioretinitis, abnormalities of the central nervous system that lead to neurologic deficiencies such as psychomotor, convulsions and mental retardation, hydrocephalus and intracranial calcifications) which differ in their severity and incidence. These data are presented in Technical Appendix Table 8 of the Annex.

Duration of illness: All outcomes of toxoplasmosis are considered to be life-long sequelae of the disease. Their duration is summarized in Technical Appendix Table 8 of the Annex.

Under-reporting: Not applicable.

Note: The study of Diza et al. (*85*) dealt with the seroprevalence of *Toxoplasma gondii* on the North of Greece and it was assumed that the seroprevalence of the parasite is the same for the whole of Greece. However, this is not the case since in rural areas and in the island of Crete the percentage of sero-positive persons appears to be higher (*89*).

Other Helminthiases

Reported cases: Based on ELSTAT data (ICD-9 codes 121, 123, 124, 127–129; BTL code 076) (Technical Appendix Table 1).

Percentage foodborne: All of the helminthic parasites included in this category of diseases (*57*) have been reported to be transmitted through food (*44,67,71,90–95*). However, it is not possible to make a precise estimate of the actual percentage of cases that are transmitted through food since this information is not available for many of the sub-categories of diseases included under this classification while the relative importance of each in this BTL code is not very clear. For some of these diseases (trichinellosis, taeniasis, anisakiasis) the percentage foodborne is mentioned to be 100% (*11, 24*) and thus all the cases under this general category of diseases can be considered to be 100% foodborne. On the other hand certain species of helminthes included in this category have also been reported to have different modes of

transmission i.e., *Strongyloides stercoralis* can enter the body through the skin (*96*) and since many of them can be found in contaminated water in pools (*97*) they can be transmitted by swimming in such waters. Finally, helminthic infections are also known to have a venereal mode of transmission (*98*). Nonetheless, the above modes of transmission are not expected to be the case for most of the species in this category and thus 90% of the cases were assumed to be foodborne. In a very conservative scenario, only 30% of the cases were assumed to be foodborne as in the case of the tapeworm *Echinococcus granulosus*.

Case fatality: A case fatality of ≈3.4% has been estimated for this group of illnesses based on the number of deaths among hospitalized cases (*13*) and the total number of hospitalizations for the period 1996–2006 (*1*).

Disability weight: Most of the helminthic intestinal infections in the Greek native population are caused by the species *Enterobius vermicularis*, *Ascaris lumbricoides*, *Strongyloides stercoralis* and *Taenia* sp. (*75,99*). However autochthonous cases of helminthic infections have been mentioned to be extremely rare in Greece and most of the reported cases of helminthiases are imported following the influx of immigrants in the country (*99*). The helminthic infection most commonly identified in the foreign population (emigrants and refugees) is ancylostomiasis (caused by Ancylostoma sp.) (*75, 99*) while other species found in foreigners are *Trichuris trichiura*, *Ascaris lumbricoides*, *Strongyloides stercoralis, Taenia* sp., *Enterobius vermicularis* and *Schistostoma mansonii* (*75*). Helminthic infections due to *Ancylostoma* sp. and *Schistostoma* sp. are part of other BTL codes (075 and 072) (*57*) while *Strongyloides stercoralis* is not transmitted through food but cutaneously (*96*) so only the rest of the species mentioned above are of relevance for this item. Though all of the remaining helminthic species are primarily associated with gastrointestinal symptoms (*100–103*) they can also be associated with sequelae such as nutrient deficiencies and anemia or even epilepsia (*100,104*). The disability weights for these illnesses and their sequelae according to Lopez et al. (*105*) range from 0–0.463. Here the upper value of this range was considered to be of relevance for the reported cases and for the underreported cases which we assumed to be related with gastrointestinal symptoms a disability weight of 0.067 was selected (*22*).

Duration of illness: The duration of the disease was assumed to be 5.67 days based on ELSTAT data (*1*). In the absence of any more closely related numbers, for the under-reported

cases the median duration of diarrhea before visiting a GP in the Netherlands (6 days) was selected (*106*).

Under reporting: This factor could also be in the range of 4.6–98.6 based on what is known for the under-reporting of protozoan parasites (*7,53*). We set the most likely value for under-reporting at the middle of this range.

VIRAL DISEASES

Acute hepatitis A

Reported cases: Based on HCIDC data (Technical Appendix Table 1).

Percentage foodborne: 8% of the total cases were considered to be food-borne which is the average of the range suggested by a recent expert study (5%–11%) (*12*).

Case fatality: A case fatality rate of 0.4% was estimated by taking into account the age of acute hepatitis A patients in Greece and the case fatality rates for different age groups as presented in the HCIDC Web site (*2*).

Disability weight: A disability weight of 0.5 was selected for cases of acute hepatitis A (*107*). Reported and underreported cases were assumed to have the same severity.

Duration of illness: The duration of acute cases of hepatitis A is considered to be 9.5 days for both reported and underreported cases according to the average duration of hospitalizations in EU countries for which information could be found in the European Health For All Database (HFA-DB) (Technical Appendix Table 9).

Under-reporting: An under-reporting factor of 2 is possible based on the seriousness of the disease (*11*) though it can be as high as 9.1 (*7*). We set the most likely value for under-reporting at the middle of this range.

DISEASES DUE TO MIXED ILL-DEFINED CAUSES

Intestinal infections due to other specified microorganism

Reported cases: Based on ELSTAT data (ICD-9 codes 007, 008; BTL code 015) (Technical Appendix Table 1).

Percentage foodborne: 36% based on the relative frequency of foodborne transmission for known pathogens (*11*). In theory looking at the microorganisms included in this category (Technical Appendix Table 10) and at the percentages foodborne mentioned for some of these in literature this figure may vary between 1 and 70%.

Case fatality: To estimate mortality due to this category of diseases data for Greece from the WHO Mortality Database were used (*13*). According to this database mortality is very low for this particular ICD code ranging between 0 and 1 cases per year for the whole country population. The upper limit was considered more realistic although it is still likely to result in under-estimating of the real situation since the quality of data in this particular source has been mentioned to be low (*108*). Given the very generic nature of this category assuming a case-fatality rate is not possible.

Disability weight: Since the reported cases correspond to patients that were hospitalized, a class 3 disability weight of 0.4 was assigned (*20*). For the under-reported cases a class 1 disability weight of 0.067 was assigned (*22*).

Duration of illness: The average hospitalization time for these diseases in Greece (3.84 days) was considered (*1*). For the under-reported cases the duration of illness before visiting a GP for *Norovirus* was considered (3.8 days) (*22*).

Under-reporting: It was estimated by multiplying two separate under-reporting factors: the first underreporting factor covers the underreporting of gastroenteritis related illnesses between hospitalizations (ELSTAT data (*1*)) and visits to physicians (HCIDC data (S. Bonovas, T. Panagiotopoulos, E. Triantafillou, pers. comm.) and the second one is used to cover the underreporting between visits to physicians for gastroenteritis and cases in the community (*35*). In this way four different underreporting factors were estimated for the period 2006–2003. For the rest of the years for which no information was available to estimate the first underreporting factor, the average of the estimated underreporting factors for the period 2005–2003 was used. In theory these under-reporting factors could be anywhere between 2 and 1562 based on what is

mentioned about the underreporting of some of the pathogens included in this category (Technical Appendix Table 10).

Ill-defined intestinal infections

Reported cases: Based on ELSTAT data (ICD-9 code 009; BTL code 016) (Technical Appendix Table 1).

Percentage foodborne: A value of 36% was selected for foodborne transmission as in cases of acute gastroenteritis due to unknown etiology (*11*). Given the fact that the nature of microorganisms in this category is not known this percentage could range between 1 and 100%.

Case fatality: The mortality rate was considered to be 1 case per year for the whole country population based again on the WHO Mortality Database (*13*) as in the previous item.

Disability weight: Since the reported cases correspond to patients that were hospitalized a class 3 disability weight of 0.4 was assigned (*20*). For the under-reported cases a class 1 disability weight of 0.067 was assigned (*22*).

Duration of illness: The average hospitalization time for these diseases in Greece (3 days) was considered (*1*). For the under-reported cases the duration of illness before visiting a GP for *Norovirus* was considered (3.8 days) (*22*).

Under-reporting: The same under-reporting rate as for the previous category was assumed. Nonetheless, the range of underreporting can vary a lot in theory given what we have so far observed regarding the range of under-reporting factors for foodborne pathogens (2–1562).

References

1. Hellenic Statistical Authority [internet]. Pireas: General Secretariat of the National Statistical Service of Greece [cited 2010 Mar 25]. http://www.statistics.gr

2. Center for Infectious Diseases Control [internet]. Marousi: Ministry of Health and Welfare: Hellenic Center for Infectious Diseases Control (HCIDC) [cited 2010 Mar 25]. http://www.keelpno.gr

3. World Health Organization. WHO Surveillance Programme for Control of Foodborne Infections and Intoxications in Europe. 8th Report 1999–2000. Country Reports: Greece. Geneva: The Organization. 1999–2000. http://www.bfr.bund.de/internet/8threport/CRs/gre.pdf

4. World Health Organization. WHO Surveillance Programme for Control of Foodborne Infections and Intoxications in Europe. 7th Report. Country Reports: Greece 1993–1998. Geneva: The Organization. 2003. http://www.bfr.bund.de/internet/7threport/CRs/GRE.pdf

5. Denny J, McLaughlin J. Human *Listeria monocytogenes* infections in Europe - An opportunity for improved European Surveillance. Euro Surveill. 2008;13:8082. PubMed

6. Mossialos E, Allin S, Davaki K. Analysing the Greek health system: A tale of fragmentation and inertia. Health Econ. 2005;14:S151–68. PubMed

7. Scallan E, Hoekstra RM, Angulo FJ, Tauxe RV, Widdowson MA, Roy SL, et al. Foodborne illness acquired in the United States - Major pathogens. Emerg Infect Dis. 2011;17:7–15. PubMed

8. The Community Summary Report on Trends and Sources of Zoonoses, Zoonotic Agents, Antimicrobial Resistance and Foodborne Outbreaks in the European Union in 2006. The EFSA Journal. 2007;5(12).

9. McManus DP, Zhang W, Li J, Bartley PB. Echinococcosis. Lancet. 2003;362:1295–304. PubMed

10. Pappas G, Papadimitriou P, Akritidis N, Christou L, Tsianos EV. The new global map of human brucellosis. Lancet Infect Dis. 2006;6:91–9. PubMed

11. Mead PS, Slutsker L, Dietz V, McCaig FL, Breese SJ, Shapiro C, et al. Food-related illness and death in the United States. Emerg Infect Dis. 1999;5:607–25. PubMed

12. Havelaar AH, Galindo AV, Kurowicka D, Cooke RM. Attribution of foodborne pathogens using structured expert elicitation. Foodborne Pathog Dis. 2008;5:649–59. PubMed

13. WHO Mortality Database [internet]. Geneva: World Health Organization [cited 2009 Mar 25]. http://www.who.int/whosis/mort/download/en/index.html

14. Budke CM, Deplazes P, Torgerson RP. Global socioeconomic impact of cystic echinococcosis. Emerg Infect Dis. 2006;12:296–303. PubMed

15. Roth F, Zinsstag J, Orkhon D, Chimed-Ochir G, Hutton G, Cosivi O, et al. Human health benefits from livestock vaccination for brucellosis: case study. Bull World Health Organ. 2003;81:867–76. PubMed

16. Van Lier EA, Havelaar AH. Disease burden of infectious diseases in Europe: a pilot study. Bilthoven: National Institute for Public Health and the Environment. 2007. Report No 215011001. http://www.rivm.nl/bibliotheek/rapporten/215011001.pdf

17. Havelaar AH, Kemmeren JM, Kortbeek LM. Disease burden of congenital toxoplasmosis. Clin Infect Dis. 2007;44:1467–74. PubMed

18. Vijgen SMC, Mangen MJJ, Kortbeek LM, van Duijnhoven YTHP, Havelaar AH. Disease burden and related costs of cryptosporidiosis and giardiasis in the Netherlands. Bilthoven: National Institute for Public Health and the Environment. 2007. http://www.rivm.nl/bibliotheek/rapporten/330081001.pdf

19. Wijewardene K, Spohr M. An attempt to measure burden of disease using disability adjusted life years for Sri Lanka. Ceylon Med J. 2000;45:110–5. PubMed

20. Murray CJL. Quantifying the burden of disease: the technical basis for disability-adjusted life years. Bull World Health Organ. 1994;72:429–45. PubMed

21. Walsh JA. Problems in recognition and diagnosis of amebiasis: estimation of the global magnitude of morbidity and mortality. Rev Infect Dis. 1986;8:228–38. PubMed

22. Kemmeren JM, Mangen MJJ, van Duynhoven YTHP, Havelaar AH. Priority setting of foodborne pathogens: disease burden and costs of selected enteric pathogens. Bilthoven: National Institute for Public Health and the Environment. 2006. 330080001. http://www.rivm.nl/bibliotheek/rapporten/330080001.pdf

23. Abgueguen P, Delbos V, Chennebault JM, Fanello S, Brenet O, Alquier P, et al. Nine cases of foodborne botulism type B in France and literature review. Eur J Clin Microbiol Infect Dis. 2003;22:749–52. PubMed

24. Vaillant V, De Valk H, Baron E, Ancelle T, Colin P, Delmas M-C, et al. Foodborne Infections in France. Foodborne Pathog Dis. 2005;2:221–32. PubMed

25. Therre H. Botulism in the European Union. Eurosurveillance. 1999;4(1).

26. Sobel J, Tucker N, Sulka A, McLaughlin J, Maslanka S. Foodborne Botulism in the United States, 1990–2000. Emerg Infect Dis. 2004;10:1606–11. PubMed

27. Mann JM, Martin S, Hoffman R, Marrazzo S. Patient recovery from type A botulism: morbidity assessment following a large outbreak. Am J Public Health. 1981 March 1, 1981;71(3):266–269.

28. Aureli P, Di Cunto M, Maffei A, De Chiara G, Franciosa G, Accorinti L, et al. An outbreak in Italy of botulism associated with a dessert made with mascarpone cream cheese. Eur J Epidemiol. 2000;16:913–8. PubMed

29. Kongsaengdao S. An outbreak of botulism in Thailand: clinical manifestations and management of severe respiratory failure. Clin Infect Dis. 2006;43:1247–56. PubMed

30. World Health Organization. Foodborne disease outbreaks: Guidelines for investigation and control. Geneva: The Organization. 2008. http://www.who.int/foodsafety/publications/foodborne_disease/fdbmanual/en/

31. Andriopoulos P, Tsironi M, Deftereos S, Aessopos A, Assimakopoulos G. Acute brucellosis: presentation, diagnosis and treatment of 144 cases. Int J Infect Dis. 2007;11:52–7. PubMed

32. National Institute for Public Health and the Environment. Our food our health. Healthy diet and safe food in the Netherlands. Bilthoven: The Institute. 2006. Report No 270555009. http://www.rivm.nl/bibliotheek/rapporten/270555009.pdf

33. Vorou R, Gkolfinapoulou K, Dougas G, Mellou K, Pierroutsakos IN, Papadimitriou T. Rapid communications. Local Brucellosis outbreak on Thassos, Greece: a preliminary report. Euro Surveill. 2008;13:1–2.

34. Bikas C. Jelastopulu, Leotsinidis M, Kondakis X. Epidemiology of human brucellosis in a rural area of north-western Peloponnese in Greece. Eur J Epidemiol. 2003;18:267–74. PubMed

35. Adak GK, Long SM, O'Brien SJ. Trends in indigenous foodborne disease and deaths, England and Wales: 1992 to 2000. Gut. 2002;51:832–41. PubMed

36. Andreoletti O, Budka H, Buncic S, Collins JD, Griffin J, Hald T, et al. EFSA Panel on Biological Hazards (BIOHAZ); Scientific opinion on quantification of the risk posed by broiler meat to human campylobacteriosis in the EU. EFSA Journal; 2010. p. 1437.

37. Haagsma JA, Siersema PD, de Wit NJ, Havelaar AH. Disease burden of post-infectious irritable bowel syndrome in the Netherlands. Epidemiol Infect. 2010;138:1650–6. PubMed

38. Havelaar AH, de Wit MAS, van Koningsveld R, van Kempen E. Health burden in the Netherlands due to infection with thermophilic *Campylobacter* spp. Epidemiol Infect. 2000;125:505–22. PubMed

39. Wheeler JG, Sethi D, Cowden MJ, Wall GP, Rodrigues CL, Tompkins SD, et al. Study of infectious intestinal disease in England: rates in the community, presenting to general practice, and reported to national surveillance. BMJ. 1999;318:1046–50. PubMed

40. Ekdahl K, Giesecke J. Travellers returning to Sweden as sentinels for comparative disease incidence in other European countries, *Campylobacter* and *Giardia* infection as examples. Eurosurveillance. 2004;9(9).

41. Ammon A. Surveillance of enterohaemorrhagic *E. coli* (EHEC) infections and Haemolytic Uraemic Syndrome (HUS) in Europe. Eurosurveillance. 1997;2(12).

42. Rangel JM, Sparling HP, Crowe C, Griffin MP, Swerdlow LD. Epidemiology of *Escherichia coli* O157–H7 outbreaks, United States, 1982–2002. Emerg Infect Dis. 2005;11:603–9. PubMed

43. Haque R, Huston CD, Hughes M, Houpt E, Petri WA. Amebiasis. N Engl J Med. 2003;348:1565–73. PubMed

44. International Commisssion on Microbiological Specifications for Foods. Microorganisms in Foods 5. Characteristics of Microbial Pathogens. London: Blackie Academic and Professional; 1996.

45. Levett PN. Leptospirosis. Clin Microbiol Rev. 2001 April 1, 2001;14(2):296–326.

46. Unz RF. Disease transmission by contaminated water. In: Nemerow LN, Agardy IF, Sullivan P, Salvato AJ, editors. Environmental Engineering: Volume 2: Prevention and Response to Water-, Food-, Soil-, and Air-Borne Disease and Illness. 6th edition ed. New York: Wiley; 2009.

47. Czachor SJ. A brief overview of food-borne illness. Antimicrobials and Infectious Diseases Newsletter. 1998;17:73–5.

48. Theocharidou D, Loga K, Papa-Konidari A. Pulmonary involvement in leptospirosis, Northern Greece. In:18th European Congress of Clinical Microbiology and Infectious Diseases, Barcelona, Spain 19–22 April 2008

49. Jansen A, Schoneberg I, Frank C, Alpers K, Schneider T, Stark K. Leptospirosis in Germany, 1962–2003. Emerg Infect Dis. 2005;11:1048–54. PubMed

50. Binder WD, Mermel LA. Leptospirosis in an urban setting: case report and review of an emerging infectious disease. J Emerg Med. 1998;16:851–6. PubMed

51. European Hospital Morbidity Database (EHMD) [internet]. Geneva: World Health Organization Regional Office for Europe [cited 2008 Oct 25]. http://data.euro.who.int/hmdb/index.php

52. Schlech WF III. Foodborne listeriosis. Clin Infect Dis. 2000;31:770–5. PubMed

53. Rocourt J, Moy G, Vierk K, Schlundt J. The present state of foodborne disease in OECD countries. Geneva: World Health Organization. 2003. http://www.who.int/foodsafety/publications/foodborne_disease/en/OECD%20Final%20for%20WEB.pdf

54. de Jong B, Ekdahl K. The comparative burden of salmonellosis in the European Union member states, associated and candidate countries. BCM Public Health. 2006;6(4).

55. Bhan MK, Bahl R, Bhatnagar S. Typhoid and paratyphoid fever. Lancet. 2005;366:749–62. PubMed

56. Kadhiravan T, Wig N, Kapil A, Kabra SK, Renuka K, Misra A. Clinical outcomes in typhoid fever: adverse impact of infection with nalidixic acid-resistant *Salmonella typhi.* BMC Infect Dis. 2005;5:37. PubMed

57. World Health Organization. Manual of the international statistical classification of diseases, injuries and causes of death [in Greek]. Athens; 1980.

58. Davies AR, Capell C, Jehanno D, Nychas GJE, Kirby RM. Incidence of foodborne pathogens on European fish. Food Contr. 2001;12:67–71.

59. Johnson D. Food-borne poisoning. Australas Emerg Nurs J. 1999;2:19–20.

60. Le Loir Y, Baron F, Gautier M. *Staphylococcus aureus* and food poisoning. Genet Mol Res. 2003;2:63–76. PubMed

61. Granum PE, Lund T. *Bacillus cereus* and its food poisoning toxins. FEMS Microbiol Lett. 1997;157:223–8. PubMed

62. Shandera WX, Tacket OC, Blake PA. Food poisoning due to *Clostridium perfringens* in the United States. J Infect Dis. 1983;147(1):167–170.

63. de Jong AEI. *Clostridium perfringens*: spores & cells, media & modelling [PhD Thesis]. Wageningen: Wageningen University; 2003.

64. Haagsma JA, van der Zanden BP, Tariq L, Van Pelt W, Van Duynhoven YTPH, Havelaar AH. Disease burden and costs of selected foodborne pathogens in the Netherlands, 2006. Bilthoven: National Institute for Public Health and the Environment. 2009. Report No 330331001. Available from: http://www.rivm.nl/bibliotheek/rapporten/330331001.pdf

65. Adams MR, Moss MO. Food Microbiology. Second ed. Cambridge: The Royal Society of Chemistry; 2006.

66. Pinto B, Chenoll E, Aznar R. Identification and typing of food-borne *Staphylococcus aureus* by PCR-based techniques. Syst Appl Microbiol. 2005;28:340–52. PubMed

67. Orlandi AP, Chu TDM, Bier WJ, Jackson JG. Parasites and the Food Supply. Scientific Status Summary. IFT. Food Technol. 2002;56:72–81.

68. Marshall MM, Naumovitz D, Ortega Y, Sterling CR. Waterborne protozoan pathogens. Clin Microbiol Rev. 1997 January 1, 1997;10(1):67–85.

69. Fodor T. Unanswered questions about the transmission of amebiasis. Bull N Y Acad Med. 1981;57:224–6. PubMed

70. Hurwitz AL, Owen LR. Venereal transmission of intestinal parasites. West J Med. 1978;128:89–91. PubMed

71. Illustrated lecture notes on Tropical Medicine [internet]. Antwerp: Antwerp Institute of Tropical Medicine [cited 2009 Feb 5]. http://content-e.itg.be/content-e/pub_ITG/Illustrated_lecture_notes_on_Tropical_Medicine_1169817124568/index.htm

72. Porter JD, Ragazzoni PH, Buchanon DJ, Waskin AH, Juranek DD, Parkin EW. *Giardia* transmission in a swimming pool. Am J Public Health. 1988;78:659–62. PubMed

73. Casemore DP. Foodborne protozoal infection. Lancet. 1990;336:1427–32. PubMed

74. Evangelopoulos A, Legakis N, Vakalis N. Microscopy, PCR and ELISA applied to the epidemiology of amoebiasis in Greece. Parasitol Int. 2001;50:185–9. PubMed

75. Papazahariadou MG, Papadopoulos EG, Frydas SE, Mavrovouniotis C, Constantinidis TC, Antoniadou-Sotiriadou K, et al. Prevalence of gastrointestinal parasites in the Greek population: local people and refugees. Annals of Gastroenterology. 2004;17:194–8.

76. Carabin H, Budke MC, Cowan DL, Willingham LA III, Torgeson RP. Methods for asessing the burden of parasitic zoonoses: echinococcosis and cysticercosis. Trends Parasitol. 2005;21:327–33. PubMed

77. Sreter T, Szell Z, Egyed Z, Varga I. *Echinococcus multilocularis*: An Emerging Pathogen in Hungary and Central Eastern Europe? Emerg Infect Dis. 2003;9:384–6. PubMed

78. Kern P, Bardonnet K, Renner E, Auer H, Pawloski Z, Ammann WR, et al. European echinococcosis registry: human alveolar echinococcosis, Europe, 1982–2000. Emerg Infect Dis. 2003;9:343–9. PubMed

79. Sotiraki S, Himonas C, Korkoliakou P. Hydatidosis-echinococcosis in Greece. Acta Trop. 2003;85:197–201. PubMed

80. Koulas SG, Sakellariou A, Betzios J, Nikas K, Zikos N, Pappas-Gogos G, et al. A 15-year experience (1988–2003) in the management of liver hydatidosis in Northwestern Greece. Int Surg. 2006;91:112–6. PubMed

81. World Health Organization. The global burden of foodborne disease: taking stock and charting the way forward: WHO consultation to develop a strategy to estimate the global burden of foodborne

diseases, Geneva, 25–27 September 2006. Geneva: The Organization. 2007. http://www.who.int/foodsafety/publications/foodborne_disease/fbd_2006.pdf

82. Avgerinos ED, Pavlakis E, Stathoulopoulos A, Manoukas E, Skarpas G, Tsatsoulis P. Clinical presentations and surgical management of liver hydatidosis: our 20 year experience. HPB (Oxford). 2006;8:189–93. PubMed

83. Antsaklis A, Daskalakis G, Papantoniou N, Mentis A, Michalas S. Prenatal diagnosis of congenital toxoplasmosis. Prenat Diagn. 2002;22:1107–11. PubMed

84. Galanakis E, Manoura A, Antoniou M, Sifakis S, Korakaki E, Hatzidaki E, et al. Outcome of toxoplasmosis acquired during pregnancy following treatment in both pregnancy and early infancy. Fetal Diagn Ther. 2007;22:444–8. PubMed

85. Diza E, Frantzidou F, Souliou E, Arvanitidou M, Gioula G, Antoniadis A. Seroprevalence of *Toxoplasma gondii* in northern Greece during the last 20 years. Clin Microbiol Infect. 2005;11:719–23. PubMed

86. Pappas G, Roussos N, Falagas ME. Toxoplasmosis snapshots: Global status of *Toxoplasma gondii* seroprevalence and implications for pregnancy and congenital toxoplasmosis. Int J Parasitol. 2009;39:1385–94. PubMed

87. Cook AJC, Gilbert RE, Buffolano W, Zufferey J, Petersen E, Jenum PA, et al. Sources of toxoplasma infection in pregnant women: European multicentre case-control study. BMJ. 2000;321:142–7. PubMed

88. Gibbs RS. The origins of stillbirth: infectious diseases. Semin Perinatol. 2002;26:75–8. PubMed

89. Antoniou M, Tzouvali H, Sifakis S, Galanakis E, Georgopoulou E, Liakou V, et al. Incidence of toxoplasmosis in 5532 pregnant women in Crete, Greece: management of 185 cases at risk. Eur J Obstet Gynecol Reprod Biol. 2004;117:138–43. PubMed

90. Cho SY, Bae J, Seo BS, Lee SH. Some aspects of human sparganosis in Korea. Kisaengchunghak Chapchi. 1975;13:60–77. PubMed

91. Fried B, Graczyk KT, Tamang L. Food-borne intestinal trematodiases in humans. Parasitol Res. 2004;93:159–70. PubMed

92. Hemsrichart V. *Ternidens deminitus* infection: first pathological report of a human case in Asia. J Med Assoc Thai. 2005;88:1140–3. PubMed

93. Pelloux H, Faure O. Toxocarose de l'adulte. Rev Med Interne. 2004;25:201–6. PubMed

94. Rusnak JM, Lucey RD. Clinical gnathostomiasis: Case report and review of the english language literature. Clin Infect Dis. 1993;16:33–50. PubMed

95. Smith HV. Detection of parasites in the environment. Parasitology. 1998;117:S113–41. PubMed

96. Schad GA, Rozeboom LE. Integrated control of helminths in human populations. Annu Rev Ecol Syst. 1976;7:393–420.

97. Totkova A, Klobusicky M, Valent M, Tirjakova E. [Helminth and protozoan findings in the water of school swimming pools]. Epidemiol Mikrobiol Imunol. 1994;43:130–6. PubMed

98. Phillips SC, Mildvan D, William DC. Sexual transmission of enteric protozoa and helminths in a venereal-disease-clinic population. N Engl J Med. 1981;305:603–6. PubMed

99. Tzanetou K. Diagnostic approach of helminthic parasitic infections with interest for clinical microbiologist. Acta Microbiologica Hellenica. 2008;53:16–31.

100. Bethony J, Brooker S, Albonico M, Geiger SM, Loukas A, Diemert D, et al. Soil-transmitted helminth infections: ascariasis, trichuriasis and hookworm. Lancet. 2006;367:1521–32. PubMed

101. Song EK, Kim IH, Lee SO. Unusual manifestations of *Taenia solium* infestation. J Gastroenterol. 2004;39:288–91. PubMed

102. Ok KS, Kim YS, Song JH, Lee JH, Ryu SH, Lee JH, et al. *Trichuris trichiura* infection diagnosed by colonoscopy: case reports and review of literature. Korean J Parasitol. 2009;47:275–80. PubMed

103. Jardine M, Kokai G, Dalzell AM. *Enterobius vermicularis* and colitis in children. J Pediatr Gastroenterol Nutr. 2006;43:610–2. PubMed

104. Praet N, Speybroek N, Manzanedo R, Berkvens D, Nforninwe DN, Zoli A, et al. The disease burden of *Taenia solium* cysticercosis in Cameroon. PLoS Negl Trop Dis. 2009;3:e406. PubMed

105. Lopez DA, Mathers DC, Ezzati M, Jamison TD, Murray CJL. 2006. Global burden of disease and risk factors. http://www.dcp2.org/pubs/GBD

106. de Wit MA, Koopmans PGM, Kortbeek ML, van Leeuwen NJ, Bartelds AIM, van Duynhoven THPY. Gastroenteritis in sentinel general practices, the Netherlands. Emerg Infect Dis. 2001;7:82–91. PubMed

107. Shuval H. Estimating the global burden of thalassogenic diseases: human infectious diseases caused by wastewater pollution of the marine environment. J Water Health. 2003;1:53–64. PubMed

108. Mathers CD, Ma Fat D, Inoue M, Rao C, Lopez DL. Counting the dead and what they died from: an assessment of the global status of cause of death data. Bull World Health Organ. 2005;83:171–7. PubMed

109. European Health for All Database (HFA-DB) [internet]. Geneva: World Health Organization Regional Office for Europe [cited 2008 Oct 30]. http://www.euro.who.int/hfadb

110. Hall G, Kirk DM, Becker N, Gregory EJ, Unicomb L, Millard G, et al. Estimating foodborne gastroenteritis, Australia. Emerg Infect Dis. 2005;11:1257–64. PubMed

Technical Appendix Table 1. Reported cases of illness that may be transmitted through food in Greece in the period 1996–2006 based on the Hellenic Centre of Infectious Diseases Control (HCIDC) and the Hellenic Statistical Authority (ELSTAT). Data are expressed as cases per million inhabitants by using population yearly figures collected from the ELSTAT. Values have been rounded to include significant digits

Diseases	**1996**	**1997**	**1998**	**1999**	**2000**	**2001**	**2002**	**2003**	**2004**	**2005**	**2006**	**Average**	**SD**
Bacterial													
Botulism	-*	-*	0	0	0	0.091	0.091	0.091	0.091	0.090	0.090	0.061	0.045
Brucellosis	73	80	102	115	102	93	66	71	58	51	61	79	21
Campylobacteriosis	-*	-*	13	28	24	-*	-*	-*	31	21	26	24	6.4
EHEC†	-*	-*	-*	-*	-*	-*	-*	0.18	0.18	0	0.090	0.11	0.087
Leptospirosis	-*	-*	1.1	2.1	1.2	1.9	1.8	4.4	2.9	3.0	1.9	2.3	1.0
Listeriosis‡	-*	-*	0.093	0.64	0.55	0.27	0.46	0.091	0.27	0.72	0.63	0.42	0.24
Salmonellosis	92	54	85	88	84	28	49	94	140	111	89	83	31
Shigellosis	32	2.2	8.5	8.6	6.7	1.1	1.5	0.73	5.8	2.4	2.8	6.6	9.0
Typhoid and paratyphoid fever	11	10	9.8	10	2.9	5.0	1.6	2.9	3.6	3.7	1.6	5.6	3.7
Food poisoning§	13	46	15	26	23	86	68	24	25	16	4.1	31	25
Parasitic													
Amoebiasis	3.2	4.2	3.8	3.0	1.7	3.0	0.73	1.6	2.3	2.9	0.81	2.5	1.2
Echinococcosis	84	84	58	66	65	56	47	48	44	43	37	57	16
Viral: acute hepatitis A	10	12	24	24	15	19	22	7.0	6.5	16	12	15	6.4
Mixed/Ill-defined causes													
Other helminthiases	2.6	2.0	2.4	3.0	3.7	6.0	4.0	3.0	4.4	0.72	3.6	3.2	1.4
Other specified microorganism§	94	45	37	23	39	41	17	45	11	30	19	37	23
Ill-defined infections	1635	1672	1790	1819	1663	1834	2266	2099	2378	2424	2595	2016	349

*Data for these years were not available.

† EHEC, enterohemorrhagic *Escherichia coli*.

‡ For the years 2001 and 2002 data from a European report on listeriosis in EU countries were used (*5*).

§Reported cases by the ELSTAT were in some instances part of very generic BTL codes (“Food poisoning” and “Intestinal infections due to other specified microorganisms”) that include illnesses due to different kinds of microorganisms some of which are reported separately by the HCIDC (campylobacteriosis, EHEC, botulism and salmonellosis) or estimated indirectly (giardiasis, cryptosporidiosis). When this kind of over-lapping was observed, the reported cases due to specified causes were subtracted from the corresponding BTL code to prevent overestimating the impact of these illnesses.

Technical Appendix Table 2. Parameters of the Pert distributions used to describe foodborne transmission, under-reporting and case fatality of foodborne illnesses

**Diseases**	Food attribution (%)			Under-reporting			Case-fatality (%)		
	min, mean, max*	Data origin†	Source	min, mean, max*	Data origin†#	Source	min, mean, max*	Data origin†#	Source
Bacterial									
Botulism	80, 100, 100	EU,EU,EU	(*23, 24*)	1.625, 1.8125, 2	EU/U.S.	(*11, 25*)	3, 10.15, 17.3	U.S./U.S.	(*7, 26*)
Brucellosis	50,84,100	U.S., GR, other	(*7, 11, 31*)	2, 10.85, 19.7	other¶	-	0.9, 2, 5	U.S./GR/U.S.	(*7, 11, 33*)
Campylobacteriosis	30, 55, 80	EU/EU‡/EU	(*12*)	7.6, 274.8, 542	UK/EU	(*39, 40*)	0.1, 0.1265, 0.153	U.S./UK	(*7, 11, 35*)
EHEC§	40, 51, 90	EU/EU/EU	(*12, 41*)	2, 14.05, 26.1	UK/U.S.	(*7, 35*)	0.25, 0.54, 0.83	U.S./U.S.	(*11, 42*)
Leptospirosis	1, 5, 49	GR,other,GR	(*48*)	10, 15, 20	other¶	(*45*)	5, 10, 15	U.S./U.S.	(*45*)
Listeriosis	69, 99, 100	EU/EU/U.S.	(*7, 12, 35*)	1.1, 1.7, 2.3	EU/U.S.	(*7, 53*)	10, 30, 44	U.S./U.S.	(*52*)
Salmonellosis	55, 95, 95	EU/EU/EU	(*12, 24*)	3.2, 51.45, 99.7	EU/EU	(*39, 54*)	0.5, 0.701, 0.902	U.S./UK	(*7, 35*)
Shigellosis	8.2, 10, 31	EU/EU/U.S.	(*7, 24, 35*)	3.4, 18.35, 33.3	EU/U.S.	(*7, 35*)	0.1, 0.13, 0.16	U.S./U.S.	(*7, 11*)
Typhoid and paratyphoid fever	55, 80, 95	EU/EU/EU	(*12, 35*)	2, 7.65, 13.3	EU/U.S.	(*7, 35*)	0.4, 0.95, 1.5	U.S./EU	(*11, 24*)
Food Poisoning	87, 100, 100	EU/EU/EU	(*12, 35*)	29.3, 185.65, 342	U.S./EU	(*7, 53*)	0, 0.025, 0.05	EU/U.S.	(*11, 64*)
Parasitic									
Amoebiasis	10, 50, 100	other¶	-	9.2, 9.6, 10	other¶	(*73*)	0.1, 0.2, 0.3	GL/GL	(*21*)
Cryptosporidiosis	5.6, 5.6, 8	EU/EU/U.S.	(*11, 35*)	7.4, 53, 98.6	EU/U.S.	(*7, 35*)	0.07, 0.335, 0.6	EU/EU	(*18, 35*)
Echinococcosis	30, 30, 100	GL/GL/other	(*81*)	2, 3, 4	other/GL	(*14*)	1, 2.24, 3	GL/GR/GR	(*14, 76, 82*)
Giardiasis	5, 10, 30	EU/EU/EU	(*12, 35*)	4.6, 25.45, 46.3	EU/U.S.	(*7, 35*)	0, 0.05, 0.1	EU/U.S.	(*7, 18*)
Toxoplasmosis	30, 50, 63	EU/EU/EU	(*24, 87*)	n.a.	-	-	3.3, 3.75, 4.8	EU/EU	(*17*)
Viral: acute hepatitis A	5, 8, 11	EU/EU‡/EU	(*12*)	2, 5.55, 9.1	other/U.S.	(*7*)	0.3, 1.35, 2.4	U.S./U.S.	(*7, 11*)
Mixed/Ill-defined causes									
Other helminthiases	30, 90, 100	other¶	-	4.6, 51.6, 98.6	EU/U.S.	(*7, 53*)	3.37	GR	(*13*)
Intestinal infections due to other specified microorganism	1, 36, 70	other¶	-	2, 402, 1562	other¶	-	**	GR	(*13*)
Ill-defined intestinal infections	1, 36, 50	other¶	-	2, 402, 1562	other¶	-	**	GR	(*13*)

*Minimum (min), most likely (mean), and maximum (max) parameters of each Pert distribution.
†Origin of the data selected to describe the minimum, most likely and maximum value of each Pert distribution; GR = Greece, EU = Europe, U.S. = United States, GL = global data, other = assumption when no other data have been found
‡The average of the range of values mentioned in European studies was selected.
§EHEC, enterohemorrhagic *Escherichia coli*.
¶No or very few data have been found so a range is considered to cover all possibilities after performing a review of existing literature on possible transmission routes or underreporting factors.
#For the under-reporting factors and the case-fatality rates the most likely value is set in the middle of the range so the origin of the data represents only the extremes of the range (minimum and maximum values) unless stated otherwise.
**For these illnesses YLL were estimated by using the yearly reported number of deaths from Greece in the WHO Mortality Database.

Technical Appendix Table 3. Disability weights related to the diseases included in the present study

Diseases	Disability weights			
	Reported or estimated cases	Source	Under-reported cases	Source
Bacterial				
Botulism				
moderate cases	0.600	(*20*)	0.600	(*20*)
severe cases	0.906	(*20*)	0.906	(*20*)
Brucellosis	0.200	(*15*)	0.200	(*15*)
Campylobacteriosis			0.067	(*38*)
gastroenteritis	0.393	(*16*)		
reactive arthritis	0.140	(*16*)		
GBS 1st year*	0.250	(*16*)		
GBS long term sequelae	0.160	(*16*)		
IBD†	0.260	(*16*)		
IBS‡	0.042	(*37*)		
EHEC§			0.067	(*38*)
watery diarrhea and hemorrhagic colitis	0.393	(*16*)		
HUS¶ and ESRD#	-**			
Leptospirosis	0.920	(*20*)	0.096	(*20*)
Listeriosis	-††		-††	
Salmonellosis			0.067	(*22*)
gastroenteritis	0.393	(*16*)		
IBD†	0.260	(*16*)		
IBS‡	0.042	(*37*)		
reactive arthritis	0.150	(*16*)		
Shigellosis	0.220	(*19*)	0.096	(*20*)
IBS‡	0.042	(*37*)		
Typhoid and paratyphoid fever	0.600	(*20*)	0.096	(*20*)
Food poisoning	0.220	(*20*)	0.067	(*20*)
Parasitic				
Amoebiasis	0.400	(*19*)	0.067	(*22*)
Cryptosporidiosis	0.393	(*18*)	0.067	(*18*)
Echinococcosis				
cured	0.200	(*14*)	0.200	(*14*)
post surgical conditions	0.239	(*14*)	0.239	(*14*)
relapse	0.809	(*14*)	0.809	(*14*)
undiagnosed	0.200	(*14*)	0.200	(*14*)
Giardiasis	0.393	(*18*)	0.067	(*18*)
Toxoplasmosis				
clinical symptoms in the 1st year of life ‡‡	0.140	(*17*)	-§§	
asymptomatic at birth, chorioretinitis later in life	0.080	(*17*)	-§§	
Viral: acute hepatitis A	0.500	(*107*)	0.500	(*107*)
Mixed/Ill-defined causes				
Other helminthiases	0.463	(*105*)	0.067	(*22*)
Intestinal infections due to other specified microorganism	0.400	(*20*)	0.067	(*20*)
Ill-defined intestinal infections	0.400	(*20*)	0.067	(*20*)

*GBS, Guillain-Barré Syndrome.
†IBD, Inflammatory Bowel Disease.
‡IBS, Irritable Bowel Syndrome.
§EHEC, enterohemorrhagic *Escherichia coli*.
¶HUS, hemolytic-uraemic syndrome.
#ESRD, end-stage renal disease.
**For HUS (including ESRD as a sequela) it is estimated that every case corresponds to 1.05 YLD (*16*).
††Not applicable. In the case of listeriosis due to its high case fatality ratio, >95% of the DALY estimates is composed of YLL (*22*) that mainly determine the burden of the disease and therefore no YLD were estimated.
‡‡Clinical symptoms in the first year of life include chorioretinitis, intracranial calcifications, hydrocephalus and CNS abnormalities that lead to neurologic deficiencies such as mental retardation.
§§Toxoplasmosis cases are estimates for the entire population. Consequently under-reporting does not apply.

Technical Appendix Table 4. Disability weights and average duration of illness for different outcomes of reported cases of campylobacteriosis according to van Lier and Havelaar (*16*) and Haagsma (*37*)

Outcome	Disability weight	Incidence	Duration (years)
Gastroenteritis	0.393	reported cases	0.027
Reactive arthritis	0.14	0.16 per reported case	0.61
GBS 1st year	0.25	0.009 per reported case	1
GBS following years (long term sequela)	0.16	0.009 per reported case	29.26
Inflammatory bowel disease	0.26	0.0035 per reported case	44.36
Irritable bowel syndrome	0.042	0.088 per reported case	5 y

Technical Appendix Table 5. Disability weights, duration of illness and incidence for different outcomes of reported cases of salmonellosis according to van Lier and Havelaar (*16*) and Haagsma (*37*)

Outcome	Disability weight	Incidence	Duration
Gastroenteritis	0.393	all reported cases	11.2 d
Inflammatory bowel disease	0.26	0.003 per reported case	50.52 y
Irritable bowel syndrome	0.042	0.088 per reported case	5 y
Reactive arthritis	0.15	0.18 per reported case	0.61 y

Technical Appendix Table 6. Incidence, disability weights and duration for different outcomes of surgery for the treatment of cystic echinococcosis (*14*)

Surgery outcome	Incidence (%)	Disability weight	Duration (years)
Cured	74.755	0.2	1
Morbidity	16.550	0.239	5
Relapse	6.452	0.809	5
Death	2.244	–	–
*Undiagnosed	10	0.2	10

*It is assumed that 10% of all cases are undiagnosed and thus do not receive medical treatment.

Technical Appendix Table 7. Number of births during the period 1996–2006 and cases of toxoplasmosis based on the percentage of seronegative women of reproductive age (~70%), the incidence of primary infection among pregnant women (0.51%) and the maternofetal transmission rate (19.4%) (*83, 85, 86*)

Year	Pregnancies/year	Cases of toxoplasmosis
1996	100718	70
1997	102038	71
1998	100894	70
1999	100643	70
2000	103274	72
2001	102282	71
2002	103569	72
2003	104420	72
2004	105655	73
2005	107545	75
2006	112042	78

Technical Appendix Table 8. Possible outcomes of congenital toxoplasmosis, incidence among patients, duration of illness and disability weights. The duration of illness has been adjusted based on the life expectancy of the Greek population. Source: Havelaar et al. (*17*)

	Incidence (%)	Disability weights	Duration (years)
Fetal loss	3	1	78.2
Clinical symptoms in the first year of life			
Chorioretinitis	14	0.17	78.2
Intracranial calcification	11.4	0.01	78.2
Hydrocephalus	1.9	0.36	78.2
CNS abnormalities*	2.7	0.36	78.2
Neonatal death	0.75	1	78.2
Asymptomatic at birth, chorioretinitis later in life	16.9	0.08	68.2

*Includes abnormalities of the central nervous system that lead to neurologic deficiencies, that is psychomotor or other neurologic deficiencies, convulsions and mental retardation.

Technical Appendix Table 9. Average duration of acute hepatitis A based on data regarding the average length of stay in hospitals for 8 EU countries in 2006 according to the HFA-DB (*109*)

Country	Duration of illness (days)
Czech Republic	16.1
Austria	6.9
Slovenia	6.1
Cyprus	7.0
Croatia	12.7
Denmark	5.5
Finland	6.6
Slovakia	15.2
Average	9.5

Technical Appendix Table 10. Microorganisms included in BTL code "015 – Intestinal infections due to other specified microorganism accompanied by the percentage foodborne and under-reported rates found for each species in literature

Disease (microorganism)	Percentage foodborne	Under-reporting factor (reference)
Protozoal infections		
Balantidium coli	–	≥10 (*73*)
Giardia lamblia	5%–30% (*12*)	≥10 (*73*)
Coccidiosis (*Isospora belli, Isospora hominis*)	–	≥10 (*73*)
Trichomoniasis	0 (not foodborne)	–
Other intestinal diseases due to protozoa	-	≥10 (*73*)
Undefined protozoal diarrhea/dysentery	-	≥10 (*73*)
Intestinal infections		
Escherichia coli	30–70 (*11*)	38 (*11*)
Arizona group of paracolon bacilli	–	–
Aerobacter aerogenes	–	–
Proteus mirabilis, P. morganii	–	–
Other defined microorganisms (staphylococcal enterocolitis)	–	–
Microbial enteritis (undefined)	–	–
Enteritis due to specified virus (adenovirus, enterovirus)	6%–10% based on what is known for *Enterovirus* (*12*) and *Astrovirus*/*Adenovirus* (*110*)	721.3 (for astrovirus) (*35*)
Other microorganisms that cannot be classified elsewhere (viral gastroenteritis and enteritis)	1%–40% (*12*) (based on what is known for *Norovirus* and *Rotavirus*)	21.5–1562 (based on data for *Norovirus* and *Rotavirus*) (*35,39*)
